# RanBP2-dependent SUMOylation of G3BP2 inhibits formation, and promotes disassembly, of stress granules

**DOI:** 10.1091/mbc.E25-03-0135

**Published:** 2026-06-10

**Authors:** Yifan E. Wang, Javid Guliyev, Sean S.J. Ihn, Gaelen Moore, Fermisk Saleh, Hyun O. Lee, Alexander F. Palazzo

**Affiliations:** ^a^Department of Biochemistry, University of Toronto, Toronto, ON, M5S 1A8, Canada; University of Alberta

## Abstract

Stress granules and processing bodies (P-bodies) are dynamic cytoplasmic ribonucleoprotein (RNP) condensates that coordinate translation inhibition, mRNA storage, and decay during cellular stress. Emerging evidence suggests that SUMOylation contributes to the regulation of these RNP granules in human cells, though the underlying mechanisms remain largely unexplored. Here, we identify RanBP2-dependent SUMOylation as a key regulator of stress granule and P-body dynamics. Cells that lack RanBP2-mediated SUMOylation show enhanced stress granule assembly and delayed disassembly in response to oxidative stress. We further demonstrate that G3BP2, a core component of stress granules, is SUMOylated in a RanBP2-dependent manner at lysine 281, and that this modification limits spontaneous stress granule formation and promotes efficient disassembly. In addition, the loss of RanBP2-mediated SUMOylation reduces P-body abundance and promotes the merging of a subset of P-bodies with stress granules during stress. Together, our findings suggest that SUMOylation may regulate RNP granules by increasing G3BP solubility, thereby limiting stress granule formation, and by maintaining the balance and segregation of stress granules and P-bodies.

## Abstract (executive summary)

- SUMOylation has been implicated in stress granule regulation, but the specific E3 ligases and substrates involved remain poorly defined.
- We show that the nuclear pore-associated SUMO E3 ligase RanBP2 directly SUMOylates the core stress granule protein G3BP2 at lysine 281, limiting spontaneous stress granule formation and promoting their timely disassembly after oxidative stress.
- Loss of RanBP2-dependent SUMOylation also reduces P-body abundance and causes aberrant merging of P-bodies with stress granules.
- These findings reveal a mechanistic link between nuclear pore-mediated SUMOylation and cytoplasmic RNP granule homeostasis, with implications for understanding viral infection and neuroinflammatory disease.

## INTRODUCTION

Cells respond to several forms of stress, such as oxidative stress and heat shock, by inhibiting translation and shuttling mRNAs and associated proteins into biomolecular condensates, known as stress granules (Anderson and Kedersha, 2006; Ivanov *et al.*, 2019). These condensates contain mRNAs, translation initiation factors, and core protein components such as Ras GTPase-activation protein-binding proteins G3BP1 and G3BP2. These molecules are thought to form multivalent weak interactions with one another to drive the assembly of biomolecular condensates. Cells that lack G3BP1 and G3BP2 fail to form stress granules in response to most stressors, including oxidative stress induced by sodium arsenite (Kedersha *et al.*, 2016).

Stress granules also contain nuclear pore proteins and nuclear transport receptors, and stress granule assembly has been shown to impact the kinetics of nucleocytoplasmic transport (Mahboubi *et al.*, 2013; Zhang *et al.*, 2018). Conversely, nuclear transport receptors, whose primary function is thought to be transporting cargoes across the nuclear pore, also play an active role in disaggregating stress granule proteins, such as FUS (Guo *et al.*, 2018; Hofweber *et al.*, 2018; Yoshizawa *et al.*, 2018), which are prone to phase separate and form amyloid fibers (Patel *et al.*, 2015). Thus, nuclear transport receptors are thought to help disassemble biomolecular condensates. Additional pathways also contribute to stress granule clearance. When stress is relieved, stress granules are disassembled by the VCP ATPase, protein chaperones, ubiquitylation, and autophagy (Buchan *et al.*, 2013; Ryu *et al.*, 2014; Walters *et al.*, 2015; Ganassi *et al.*, 2016; Mateju *et al.*, 2017; Chitiprolu *et al.*, 2018; Tolay and Buchberger, 2021). Intriguingly, recent reports suggest that SUMOylation also contributes to stress granule disassembly (Keiten-Schmitz *et al.*, 2020; Marmor-Kollet *et al.*, 2020; Verde *et al.*, 2025; Yuan *et al.*, 2025).

RanBP2, also known as Nup358, is a SUMO E3 ligase and one of the major components of the cytoplasmic filaments of the nuclear pore complex (Bley *et al.*, 2022; Palazzo *et al.*, 2022). Besides residing at the nuclear pore, a fraction of RanBP2 localizes to cytoplasmic foci, which may either represent annulate lamellae and/or ribonucleoprotein (RNP) granules (Mavlyutov *et al.*, 2002; Sahoo *et al.*, 2017; Hampoelz *et al.*, 2019; Palazzo *et al.*, 2022; Kalarikkal *et al.*, 2024). RanBP2 has been observed in stress granules (Zhang *et al.*, 2018; Marmor-Kollet *et al.*, 2020) and has been shown to interact with G3BP2 (Ashikari *et al.*, 2017). It has also been observed in foci adjacent to both stress granules and processing bodies (P-bodies) (Sahoo *et al.*, 2017). Similar to stress granules, P-bodies are RNA-protein condensates that increase in number in response to cellular stress such as sodium arsenite treatment (Luo *et al.*, 2018). However, unlike stress granules, P-bodies are constitutively present in most cells even under unstressed conditions. Intriguingly, the depletion of RanBP2 has been shown to reduce the number of P-bodies in cells (Sahoo *et al.*, 2017) and inhibit arsenite-induced P-body formation (Ohn *et al.*, 2008), but it remains unclear whether RanBP2 affects stress granule assembly or disassembly.

In this article, we investigate the role of RanBP2-mediated SUMOylation in the dynamics of RNP condensates, with a focus on stress granules and P-bodies.

## RESULTS

### RanBP2-dependent SUMOylation delays stress granule assembly and promotes stress granule disassembly

**FIGURE 1:**
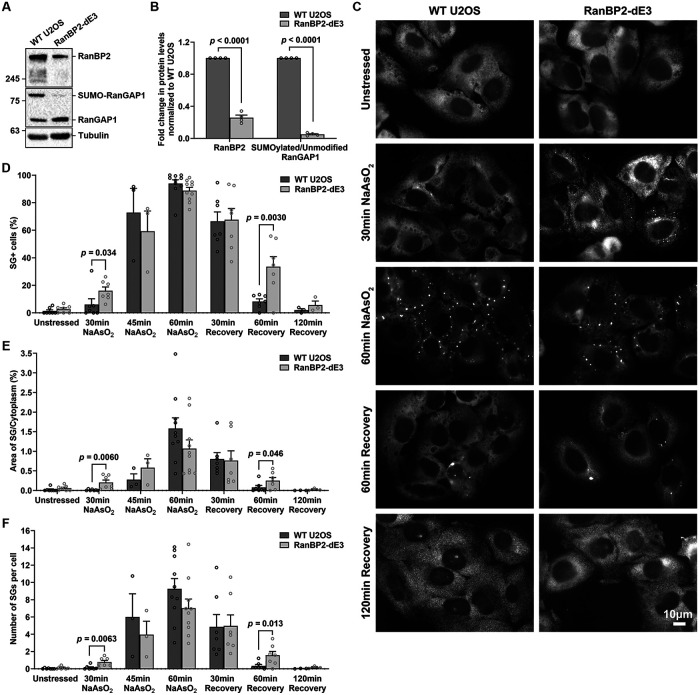
RanBP2-dependent SUMOylation delays stress granule assembly and promotes stress granule disassembly. (A and B) Lysates from unmodified (WT) or RanBP2-dE3 U2OS cell lysates were analyzed by immunoblotting for the indicated proteins (A). The levels of RanBP2, SUMO-RanGAP1, unmodified RanGAP1, and Tubulin were quantified using densitometry analysis. The ratios of RanBP2/Tubulin or SUMOylated/unmodified RanGAP1 were normalized to that of WT U2OS and plotted, with each bar representing the averages ± SEM of four independent experiments (B). (C) WT and RanBP2-dE3 U2OS cells were treated with 100 µM sodium arsenite for up to 60 min, and allowed to recover in arsenite-free media for up to 120 min. At various time points, the cells were fixed and immunostained for G3BP1/2 as a stress granule marker. Representative images for G3BP1/2 from some of the time points are shown; scale bar, 10 µm. (D–F) For images taken in C, the percentage of cells displaying stress granules (D), the percentage area of the cytoplasm occupied by stress granules (E), and the number of stress granules per cell (F) were quantified by CellProfiler. Each bar represents the averages ± SEM of more than three independent experiments.

Previously, it had been reported that inhibiting SUMOylation impairs stress granule assembly and reduces the rate of stress granule disassembly (Keiten-Schmitz *et al.*, 2020; Marmor-Kollet *et al.*, 2020; Yuan *et al.*, 2025). We took advantage of a human osteosarcoma (U2OS) cell line, where we used CRISPR-Cas9 to disrupt the SUMO E3 ligase domain of RanBP2 (Shen *et al.*, 2021). These defective E3 cells (hereafter referred to as RanBP2-dE3) still produce RanBP2, though its levels vary substantially (Figure 1, A and B) due to the fact that the modifications to the RanBP2 protein sequence slightly destabilize it (Shen *et al.*, 2021). Note that these cells contain very little SUMO-RanGAP1 (Figure 1, A and B), demonstrating that RanBP2-dependent SUMOylation is mostly abolished.

**FIGURE 2:**
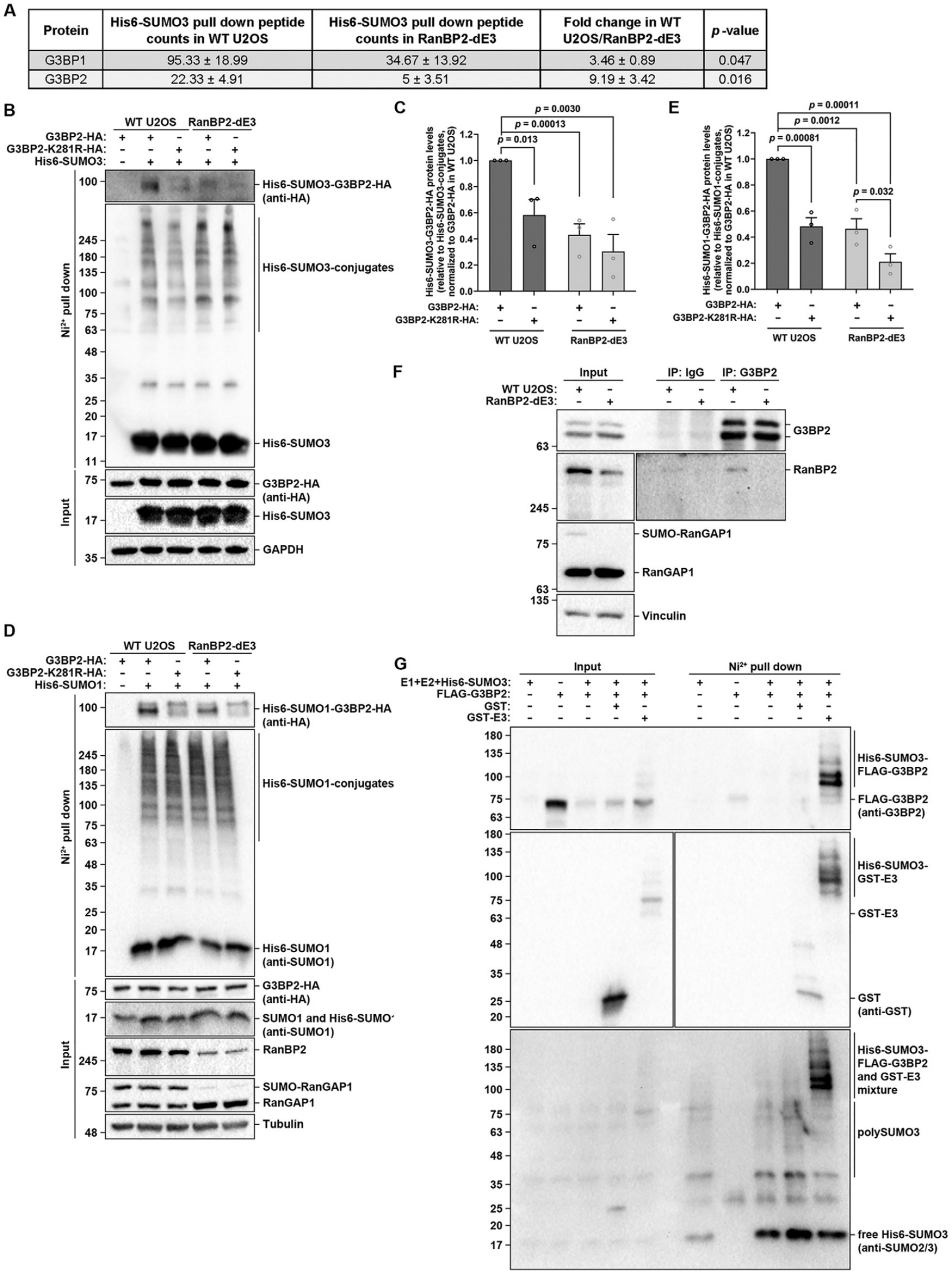
RanBP2 SUMOylates G3BP2 at lysine 281. (A) Unmodified (WT) and RanBP2-dE3 U2OS cells were transfected with a plasmid expressing His-tagged SUMO3 (His6-SUMO3) for 48 h. The His6-SUMO3-conjugates were isolated on a nickel column and analyzed by mass spectrometry. The peptide counts for G3BP1 and G3BP2 listed were averages ± SEM from three experiments. Fold change was calculated for each experiment as the ratio of peptide counts in WT U2OS to RanBP2-dE3, and shown as the average ± SEM from three experiments. (B and C) WT and RanBP2-dE3 U2OS cells were transfected with *His6-SUMO3, G3BP2-HA*, or *G3BP2-K281R-HA* as indicated. Twenty-four hours after transfection, cells were lysed under denaturing conditions, and proteins covalently attached to His6-SUMO3 were isolated by nickel column (Ni^2+^ pull down), and analyzed by immunoblotting with anti-HA for G3BP2-HA and anti-His for free and total His6-SUMO3-conjugates. Cell lysates were also directly analyzed as Input for the indicated proteins (B). The levels of G3BP2 and total His6-SUMO3-conjugates in the isolates were quantified by densitometry analysis, and the ratios of SUMOylated G3BP2-HA/total His6-SUMO3-conjugates were normalized to that of WT U2OS transfected with *G3BP2-HA* and plotted (C), with each bar representing the averages ± SEM from three independent experiments. (D and E) As in B and C, except that cells were transfected with *His6-SUMO1* instead of *His6-SUMO3*. Note that anti-SUMO1 was used for free and total His6-SUMO1-conjugates. (F) WT and RanBP2-dE3 cells were lysed and either directly analyzed as Input, or subjected to immunoprecipitation with either a control antibody (IP: IgG) or an anti-G3BP2 antibody (IP: G3BP2), followed by immunoblotting. (G) Similar to B, except that the nickel purification was performed on *E. coli* BL21 (DE3) cells transformed with plasmids expressing His6-SUMO3, SUMO E1 and E2 enzymes, FLAG-tagged G3BP2, GST control, or GST-tagged RanBP2 E3 domain fragment (GST-E3) as indicated. Protein expression was induced with 0.01 mM IPTG at 16°C for 4 h. Note that SUMOylated FLAG-G3BP2 was detected only in the presence of GST-E3, which also exhibited auto-SUMOylation.

**FIGURE 3:**
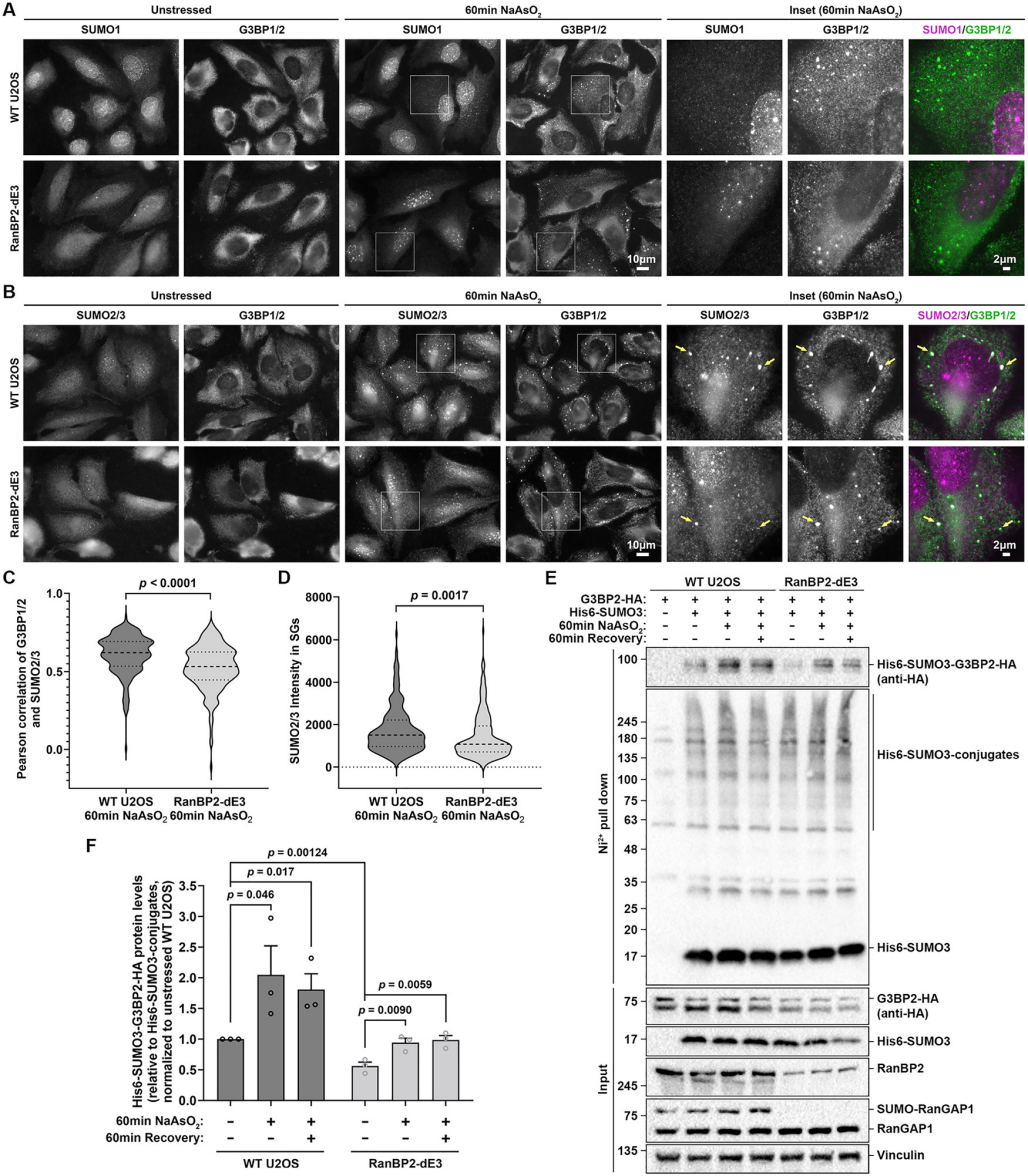
SUMO2/3 colocalizes with stress granules, and G3BP2 SUMOylation increases upon arsenite stress. (A and B) Unmodified (WT) and RanBP2-dE3 U2OS cells were either directly fixed in ice-cold methanol or treated with 100 µM sodium arsenite for 60 min before fixation, followed by immunostaining for SUMO1 (A) or SUMO2/3 (B) and G3BP1/2 as a stress granule marker. Each row represents a single field of view. The areas for the insets are indicated with white boxes, and representative stress granules are highlighted with yellow arrows. Scale bar: whole cell, 10 µm; insets, 2 µm. (C and D) Individual cytoplasmic G3BP1/2 foci in B were analyzed for colocalization with SUMO2/3 by Pearson correlation analysis (C). The fluorescence intensity of SUMO2/3 within the individual G3BP1/2 foci was quantified (D). At least 200 foci were analyzed per condition across three independent experiments. (E and F) WT and RanBP2-dE3 U2OS cells were transfected with *His6-SUMO3 and G3BP2-HA* as indicated. Twenty-four hours post-transfection, the transfected cells were treated with 100 µM sodium arsenite for 60 min, and allowed to recover in arsenite-free media for another 60 min. Cells were lysed under denaturing conditions, and proteins covalently attached to His6-SUMO3 were isolated by nickel column (Ni^2+^ pull down), and analyzed by immunoblotting with anti-HA for G3BP2-HA and anti-His for free and total His6-SUMO3-conjugates. Cell lysates were also directly analyzed as Input for the indicated proteins (E). The levels of G3BP2 and total His6-SUMO3-conjugates in the isolates were quantified by densitometry analysis, and the ratios of SUMOylated G3BP2-HA/total His6-SUMO3-conjugates were normalized to that of unstressed WT U2OS transfected with *G3BP2-HA* and plotted (F), with each bar representing the averages ± SEM from three independent experiments.

To monitor stress granule dynamics, we treated unmodified wild-type (WT) U2OS and RanBP2-dE3 cells with sodium arsenite for 60 min to induce oxidative stress and trigger stress granule assembly, and then washed out the compound to allow for cell recovery and stress granule disassembly. We fixed and stained these cells with an antibody against G3BP1/2 (Figure 1C) and analyzed stress granules using three separate metrics: 1) the percentage of cells displaying stress granules (Figure 1D), 2) the percentage area of the cytoplasm occupied by stress granules (Figure 1E), and 3) the number of stress granules per cell (Figure 1F). We observed that stress granule assembly was modestly accelerated in the RanBP2-dE3 cells, particularly at 30 min of arsenite treatment, whereas disassembly was significantly delayed, especially at 60 min of recovery. By 120 min of recovery, stress granules were fully disassembled in both cell lines, indicating that though disassembly was slightly delayed, it could still occur.

Together, these results suggest that RanBP2-SUMOylation modulates stress granule dynamics by limiting assembly and facilitating timely disassembly.

### RanBP2 SUMOylates G3BP2 on lysine 281

Efficient stress granule disassembly has been shown to require FMR1 SUMOylation (Marmor-Kollet *et al.*, 2020), but whether other stress granule components are similarly regulated remains unclear. Several SUMO proteome mass spectrometry surveys have documented G3BP1 SUMOylation under stress conditions, and G3BP2 SUMOylation under both basal and stress conditions (Hendriks *et al.*, 2014, 2017, 2018; Tammsalu *et al.*, 2014; Lamoliatte *et al.*, 2017). Recent work further suggests that arsenite-dependent SUMOylation of G3BP1 and G3BP2 is, at least in part, mediated by the TRIM28 SUMO E3 ligase (Yuan *et al.*, 2025).

We therefore asked whether RanBP2 also contributes to G3BP1 and/or G3BP2 SUMOylation. When we isolated His6-tagged SUMO3 (His6-SUMO3)-conjugated proteins from cell lysates and analyzed them by mass spectrometry, we found that G3BP1 and G3BP2 were reproducibly enriched in unmodified U2OS cells relative to RanBP2-dE3 cells (Figure 2A). Because the fold enrichment was greater and more statistically significant for G3BP2, we focused the rest of our analyses on this protein.

In previous mass spectrometry analyses, only one site, lysine 281 on G3BP2, was consistently observed to be SUMOylated. This same residue received the highest score in G3BP2 from GPS-SUMO, a SUMOylation site prediction algorithm (Zhao *et al.*, 2014) (Supplemental Figure S1). This lysine and its surrounding residues match both the consensus and inverted consensus SUMOylation motifs and are highly conserved in mammals. Note that a similar motif is also present in G3BP1, which has also been reported to be SUMOylated by TRIM28 (Yuan *et al.*, 2025).

To confirm that G3BP2 is SUMOylated in a RanBP2-dependent manner and to determine whether lysine 281 is the modified site, we coexpressed either G3BP2-HA or G3BP2-K281R-HA (where lysine 281 was replaced with arginine) and His6-tagged SUMO3 in unmodified and RanBP2-dE3 U2OS cells. We collected the lysates under denaturing conditions, isolated proteins covalently conjugated to His6-SUMO3 by nickel affinity purification, and probed for the presence of G3BP2 in the eluate by HA immunoblot. We detected G3BP2-HA in the precipitates from U2OS lysates in a His6-SUMO3-dependent manner, suggesting that G3BP2 is modified by SUMO3 in these cells (Figure 2B, quantification is shown in C). In agreement with our mass spectrometry results, we detected more His6-SUMO3-G3BP2-HA in unmodified U2OS cells compared with RanBP2-dE3 cells, and this was dependent on lysine 281 (Figure 2, B and C). Despite this, we consistently detected low levels of His6-SUMO3-G3BP2-HA in RanBP2-dE3 cells above background (Figure 2B, compare lane 1 with 4), and this was independent of lysine 281. This was unlikely to be unmodified G3BP2-HA, which migrates faster (∼75kDa) than SUMO3-conjugated G3BP2-HA (∼100kDa, Figure 2B), indicating that G3BP2 contains additional SUMOylation sites that are independent of RanBP2. We obtained similar results when we expressed His6-SUMO1 in cells (Figure 2, D and E). These results suggest that lysine 281 of G3BP2 could be modified by SUMO1 and SUMO3 (and likely SUMO2, which differs from SUMO3 by three amino acids) in a RanBP2-dependent manner.

To determine whether RanBP2 interacts with G3BP2, we immunoprecipitated endogenous G3BP2 from U2OS cell lysates and probed for RanBP2 in the eluate. Interestingly, we found that G3BP2 co-immunoprecipitated WT full-length RanBP2, but not RanBP2-dE3 mutant that lacks the E3 ligase domain (Figure 2F). Although the RanBP2-dE3 mutant is expressed at lower levels, we were unable to detect it in the immunoprecipitates even when the blots were overexposed, suggesting that the interaction between G3BP2 and RanBP2 requires the E3 domain.

To test whether RanBP2 directly SUMOylates G3BP2, we used a recombinant SUMOylation system in *Escherichia coli* BL21 cells (Weber *et al.*, 2014), where we coexpressed FLAG-G3BP2, a GST-tagged RanBP2 E3 domain fragment (GST-E3), and a plasmid that simultaneously expresses the SUMO E1 and E2 enzymes together with His6-SUMO3. As in the mammalian system, His6-SUMO3-conjugated proteins were isolated by nickel affinity purification under denaturing conditions, and the putative protein substrates in the eluates were analyzed by immunoblot. We detected robust SUMOylation of FLAG-G3BP2 in the presence of E1, E2, His6-SUMO3, and GST-E3, but not when only E1, E2, and His6-SUMO3 were expressed alone or when GST was expressed in place of GST-E3 (Figure 2G, see the anti-G3BP2 blot). Note that we also detected auto-SUMOylation of the GST-tagged RanBP2 E3 domain fragment (Figure 2G, see anti-GST blot), consistent with previous reports (Pichler *et al.*, 2002).

From these experiments, we conclude that RanBP2 can associate with and directly SUMOylates G3BP2 on lysine 281.

### SUMO2/3 localizes to stress granules

We next tested whether SUMO proteins are localized to stress granules and whether this depends on RanBP2 SUMO E3 activity. In unstressed cells, immunostaining with an antibody against either SUMO1 or SUMO2/3 (which differ by only three amino acids) showed a relatively diffuse staining pattern, with most SUMO-positive foci localized to the nucleus (Figure 3, A and B). When stress granules were induced by sodium arsenite treatment, we observed colocalization of SUMO2/3 with G3BP1/2 in unmodified U2OS cells, while SUMO1 remained mostly nuclear (Figure 3, A and B). The localization of SUMO2/3 to stress granules was also seen in RanBP2-dE3 cells (Figure 3B), but was reduced, as measured by Pearson correlation analysis and total intensity measurements (Figure 3, C and D). Note that for these analyses, cells were fixed with ice-cold methanol instead of paraformaldehyde since the latter has been reported to interfere with the detection of SUMO proteins in certain condensates (Verde *et al.*, 2025).

These data indicate that SUMO2/3 but not SUMO1 accumulates in stress granules, and this is partially dependent on RanBP2 SUMO E3 ligase activity.

### G3BP2 SUMOylation increases upon arsenite treatment

Next, we assessed how RanBP2-dependent SUMOylation of G3BP2 changes during oxidative stress and recovery, with a focus on SUMO3-conjugation given its colocalization with stress granules. Treatment of unmodified U2OS cells with sodium arsenite for 60 min led to an increase in G3BP2-HA SUMOylation relative to untreated control (Figure 3E, quantification is shown in F). After the cells were allowed to recover from arsenite treatment for 60 min, the levels of SUMOylated G3BP2-HA remained relatively constant (Figure 3, E and F). Note that throughout the time course, the total levels of G3BP2-HA did not appear to change (see input in Figure 3E). Likewise, neither arsenite stress nor recovery affected the levels of untagged endogenous G3BP2 (Supplemental Figure S2). In RanBP2-dE3 cells, the levels of SUMOylated G3BP2-HA followed a similar trend but were consistently lower when compared with unmodified cells (Figure 3, E and F). This is consistent with our earlier findings that, in addition to RanBP2, other SUMO E3 ligases likely contribute to G3BP2 SUMOylation in these cells (Figure 2, B–E).

From these experiments, we conclude that G3BP2 SUMOylation increases during oxidative stress and persists during recovery, and this SUMOylation is partially dependent on RanBP2.

### Cells expressing SUMOylation-deficient G3BP2 have accelerated assembly and decreased disassembly of stress granules

**FIGURE 4:**
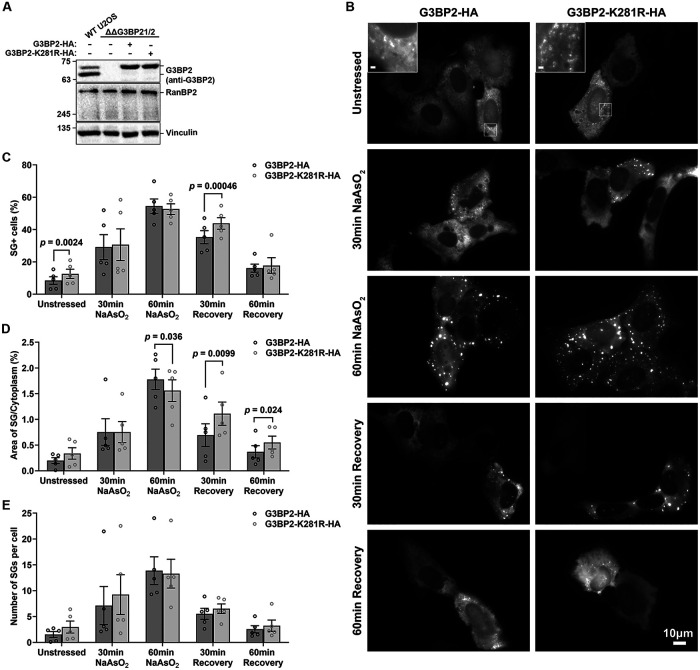
SUMOylation-deficient G3BP2 has a higher propensity to trigger stress granule assembly and is deficient in stress granule disassembly. (A) Unmodified (WT) and ΔΔG3BP1/2 U2OS cells were transfected with plasmids expressing G3BP2-HA or G3BP2-K281R-HA for 24 h, and cell lysates were analyzed by immunoblotting for the indicated proteins. (B) ΔΔG3BP1/2 U2OS cells were transfected with *G3BP2-HA* or *G3BP2-K281R-HA* for 24 h, treated with 100 µM sodium arsenite for up to 60 min, and allowed to recover in arsenite-free media for up to 60 min. At various time points, the cells were fixed and immunostained for HA to identify transfected cells and use as a stress granule marker. Representative images for G3BP2-HA from each time point are shown. Boxed areas are shown in greater magnification as insets. Scale bar: whole cell, 10 µm; insets, 1 µm. (C–E) For images taken in B, the percentage of cells displaying stress granules (C), the percentage area of cytoplasm occupied by stress granules (D), and the number of stress granules per cell (E) were quantified by CellProfiler. Each bar represents the averages ± SEM of five independent experiments.

Because cells deficient in RanBP2-dependent SUMOylation have altered stress granule dynamics in response to oxidative stress, we asked whether this is partially due to the SUMOylation of G3BP2. Given that only a small fraction of G3BP2 (and likely G3BP1) is SUMOylated, we reasoned that phenotypic effects would be most apparent if we abolished SUMOylation of all G3BP1/2 molecules in cells. We therefore used U2OS cells that lack both G3BP1 and G3BP2 (ΔΔG3BP1/2) (Kedersha *et al.*, 2016), and reintroduced either WT G3BP2-HA or SUMO-deficient mutant G3BP2-K281R-HA, which shows reduced SUMOylation (Figure 2, B–E). In these cells, exogenous G3BP2-HA protein served as the only source of G3BP1/2 activity, thus allowing us to control the SUMOylation status of the entire pool of cellular G3BP1/2. Note that the expression levels of exogenous G3BP2-HA were comparable between the two constructs and were similar to the levels of endogenous G3BP2 in WT U2OS cells (Figure 4A).

We then subjected these cells to oxidative stress and recovery (Figure 4B, stress granule metrics shown in C–E). Cells expressing the K281R mutant showed slightly altered stress granule dynamics compared with those expressing WT G3BP2-HA. In particular, at 30 min of recovery, a greater fraction of K281R-expressing cells retained stress granules, which also occupied a larger proportion of the cell area. This is consistent with our observation that RanBP2-mediated SUMOylation promotes efficient stress granule disassembly (Figure 1, C–F).

Notably, under unstressed conditions, we observed low levels of stress granule formation in cells expressing either form of G3BP2-HA, and these structures were generally small in size (see insets in Figure 4B). Although stress granules are typically absent in unstressed cells, overexpression of G3BP1 and G3BP2 alone can promote their formation (Tourrière *et al.*, 2023). Interestingly, a higher percentage of K281R-expressing cells formed spontaneous stress granules compared with those expressing G3BP2-HA (see insets in Figure 4B and quantifications in C–E). Though the fraction of unstressed cells with stress granules varied greatly between experiments (3–21%), pairwise comparison consistently showed increased stress granule formation in cells expressing the K281R mutant (Supplemental Figure S3A). These small stress granule-like structures were enriched for Caprin and PABP but lacked TIA1 (Supplemental Figure S3, B–D).

### Cells deficient in RanBP2-dependent SUMOylation have fewer P-bodies and altered stress granule–P-body organization

**FIGURE 5:**
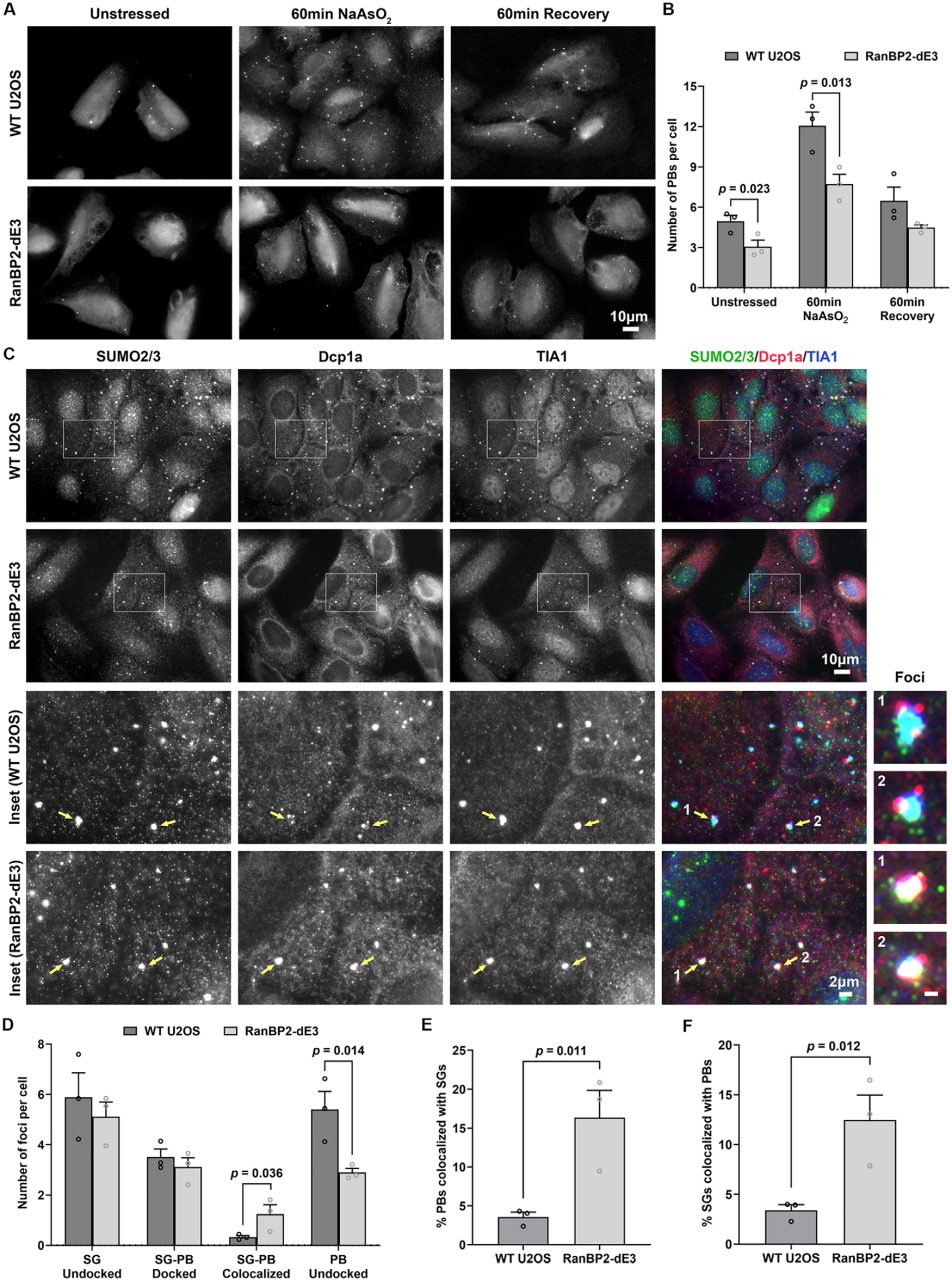
RanBP2-dependent SUMOylation promotes P-body formation and is important for P-body-stress granule organization. (A) Unmodified (WT) and RanBP2-dE3 U2OS cells were treated with 100 µM sodium arsenite for 60 min, and allowed to recover in arsenite-free media for 60 min. Cells were fixed and immunostained for Dcp1a as a P-body marker. Representative images for Dcp1a are shown; scale bar, 10 µm. (B) For images in A, the number of P-bodies per cell was quantified manually. Each bar represents the averages ± SEM of three independent experiments. (C) WT and RanBP2-dE3 U2OS cells were treated with 100 µM sodium arsenite for 60 min and fixed in ice-cold methanol. Cells were immunostained for SUMO2/3, Dcp1a (P-body marker), and TIA1 (stress granule marker). Each row represents a single field of view. The areas for the insets are indicated with white boxes, and representative stress granule–P-body docking or colocalization events are highlighted with yellow arrows. Two foci from each inset are further magnified on the right. Note that in the WT U2OS cells, P-bodies (red) are docked onto SGs (stained by SUMO2/3, green, and TIA1, blue), while in RanBP2-dE3 cells, all three markers colocalize. Scale bar: whole cell, 10 µm, insets = 2 µm; foci, 0.5 µm. (D–F) For images taken in C, the number of undocked stress granules or P-body (not in contact with one another), docked stress granule–P-body (in direct contact with each other or partially overlap), and colocalized stress granule–P-body (fully overlap or one encompassing the other) in each cell were quantified in D. The percentage of P-bodies colocalized with stress granules (E) and the percentage of stress granules colocalized with P-bodies (F) were determined by dividing the number of colocalization events by the total number of P-bodies or stress granules, respectively. Each bar represents the averages ± SEM of three independent experiments.

Stress granules interact with and share components with P-bodies (Ivanov *et al.*, 2019), and it has been reported that RanBP2 depletion leads to a decrease in both steady-state levels (Sahoo *et al.*, 2017) and arsenite-triggered proliferation (Ohn *et al.*, 2008) of P-bodies. To validate these observations and determine whether they are dependent on RanBP2-mediated SUMOylation, we immunostained unmodified and RanBP2-dE3 U2OS cells with an antibody against Dcp1a, a canonical P-body marker. Compared with unmodified U2OS cells, we found that RanBP2-dE3 cells had a statistically significant reduction in P-body number under basal and arsenite-stressed conditions, and a modest reduction during recovery (Figure 5, A–B). Thus, our results suggest that the previously reported effects are likely due to changes in RanBP2-dependent SUMOylation.

These observations prompted us to examine whether SUMO2/3-conjugated proteins localize to P-bodies, similar to what we observed with stress granules (Figure 3B). In unmodified cells, SUMO2/3 did not colocalize with the P-body marker Dcp1a under unstressed conditions (data not shown) or after arsenite treatment (Figure 5C). Instead, we observed that P-bodies frequently docked with SUMO2/3-positive foci that were also positive for TIA1, a stress granule marker (Figure 5C, see insets and foci close-up images). This is consistent with previous reports showing that P-bodies often dock on the surface of stress granules, which may facilitate the transfer of mRNAs and proteins between these two structures (Kedersha *et al.*, 2005). In contrast, in RanBP2-dE3 cells treated with arsenite, a small fraction of granules contained Dcp1a, SUMO2/3, and TIA1, indicating overlap between P-bodies and stress granules (Figure 5C, see insets and foci close-up images, for quantifications see Figure 5, D–F). Such overlap was rarely observed in unmodified cells (Figure 5, C–F). Although the number of P-bodies docked with stress granules was not significantly altered, the number of undocked P-bodies was reduced in RanBP2-dE3 cells. When the cells recovered for 60 min after arsenite treatment, these overlapping structures were barely detected in either cell line (Supplemental Figure S4), suggesting that this alteration in RNP condensates is temporary. Together, these observations suggest that RanBP2-dependent SUMOylation may play a role in maintaining the distinction between stress granules and P-bodies during arsenite stress.

It has been previously observed that ΔΔG3BP1/2 U2OS cells have lower levels of P-bodies than unmodified cells (see Figure 2 and Supplemental Figure S3 in Riggs *et al.*, [2024]), but this has not been documented in a controlled and rigorous manner. Indeed, we saw that ΔΔG3BP1/2 U2OS cells had few to any P-bodies in unstressed conditions; however, expression of either G3BP2-HA or the SUMO-deficient mutant K281R did not rescue P-body formation (Supplemental Figure S5). Interestingly, arsenite treatment induced robust P-body formation in these cells. Expression of G3BP2-HA, regardless of whether it can be SUMOylated, had little effect on P-body abundance (Supplemental Figure S5). In addition, we did not observe any overlap between P-bodies and stress granules in these cells (data not shown). These results indicate that while the loss of G3BP1/2 may impair basal P-body formation, it does not prevent stress-induced P-body assembly. It remains unclear why G3BP2-HA expression had little effect on P-body formation in ΔΔG3BP1/2 U2OS cells. This may require G3BP1 expression and may indicate differences between G3BP1 and G3BP2.

## DISCUSSION

In this study, we demonstrate that RanBP2-dependent SUMOylation regulates the dynamics of RNP granules, where it promotes stress granule clearance, facilitates P-body formation, and maintains the separation between stress granules and P-bodies. Although it remains possible that the effects that we observe are due to changes in RanBP2 levels, or that the mutations in RanBP2 disrupt RanGAP1 localization to the nuclear pore, we favor the idea that the key change is SUMOylation per se, given that we show that RanBP2 directly SUMOylates G3BP2.

We show that RanBP2-dependent SUMOylation delays stress granule formation and promotes efficient stress granule disassembly during the recovery from arsenite treatment. Although the effect on stress granule dissolution is modest, similar magnitudes have been reported when other stress granule disassembly pathways are inhibited in human cells (Ganassi *et al.*, 2016; Keiten-Schmitz *et al.*, 2020). This likely reflects the presence of multiple redundant pathways that operate in conjunction to dismantle stress granules.

Our data indicate that RanBP2 directly SUMOylates G3BP2 and that this modification may reduce its propensity to form biomolecular condensates. We show that G3BP2 is SUMOylated under basal conditions, and that SUMOylation increases during stress. Of note, because SUMO-conjugates are often low in abundance and highly transient, we overexpressed His6-SUMO and G3BP2-HA in our assays to facilitate detection of SUMOylated G3BP2 under denaturing conditions. We can detect SUMOylation of endogenous G3BP2 only when the E2 (Ubc9) is overexpressed (Supplemental Figure S6). Because SUMOylation is very labile and tends to be temporally transient, this is commonly seen with other SUMOylated proteins.

G3BP2 has two spliced isoforms (Liboy-Lugo *et al.*, 2024), as can be seen in several immunoblots (see Figures 2F and 4A; Supplemental Figures S2 and S6). Both isoforms contain a SUMOylation site within the consensus motif at lysine 281 (corresponding to lysine 248 in the shorter isoform, which lacks amino acids 243-275). Importantly, we were able to detect SUMOylation of only the larger isoform of endogenous G3BP2 (Supplemental Figure S6). Our failure to detect SUMOylation of the shorter isoform is consistent with the observation that this isoform lacks part of the region required for RanBP2-binding (amino acids 224–252), as documented by other groups (Ashikari *et al.*, 2017), though this will require further investigation.

It is likely that SUMOylation acts in a coordinated manner across multiple substrates to promote stress granule disassembly. Several stress granule proteins are found to be SUMOylated, such as FUS, TDP-43, and FMR1 (Keiten-Schmitz *et al.*, 2020; Marmor-Kollet *et al.*, 2020; Verde *et al.*, 2025). More recently, TRIM28 has been reported to SUMOylate G3BP1 at lysine 287 and G3BP2 at lysine 281 in response to stress (Yuan *et al.*, 2025). Consistent with this, our assays indicate that G3BP2 can undergo RanBP2-independent SUMOylation (Figures 2, B–D and 3, E and F), and our mass spectrometry analysis detected G3BP1 SUMOylation that was less reliant on RanBP2 (Figure 2A). Interestingly, although the earlier version of GPS-SUMO predicted K281 in G3BP2 as the primary SUMOylation site, an updated version (Gou *et al.*, 2024) predicts additional lysine residues in G3BP1 and G3BP2 as high-confidence SUMOylation sites. Whether these sites are modified in a RanBP2-dependent manner and how they affect stress granule dynamics will need to be tested in future experiments.

Although we favor a model where SUMOylation modulates the propensity of G3BP2 to form condensates, it is possible that SUMOylated G3BP2 is targeted for degradation via the SUMO-targeted ubiquitin ligase (StUbL) pathway. Indeed, the StUbL E3 ligase RNF4 has been implicated in stress granule disassembly (Keiten-Schmitz *et al.*, 2020). Despite this, we do not detect a drastic decrease in G3BP2 levels during stress or recovery, and for both overexpressed and endogenous G3BP2. It remains possible that only a small subset of G3BP2 is targeted for decay.

We also show that inhibition of RanBP2-dependent SUMOylation causes a drop in P-bodies, consistent with previous reports (Ohn *et al.*, 2008; Sahoo *et al.*, 2017). This phenotype does not appear to depend on G3BP2 SUMOylation, as neither the presence nor absence of G3BP2 proteins (either WT or the K281R mutant) affects P-body number in ΔΔG3BP1/2 U2OS cells (Supplemental Figure S5). Nevertheless, in RanBP2-dE3 cells, we observe a population of granules containing both P-body and stress granule markers, suggesting that RanBP2-mediated SUMOylation helps prevent these two structures from merging. It is also interesting that RanBP2-dependent SUMOylation appears to have opposing effects on these two condensates, promoting P-body formation while limiting stress granule assembly. Given that stress granules and P-bodies share several components (Youn *et al.*, 2018), and our data showing the temporary merging of some of these structures in a RanBP2-dependent manner (Figure 5, C–F), it is possible that SUMOylation regulates the exchange of proteins and mRNAs between these two compartments.

There appears to be much cross-talk between the nuclear pore and stress granules (Mahboubi *et al.*, 2013; Guo *et al.*, 2018; Hofweber *et al.*, 2018; Yoshizawa *et al.*, 2018; Zhang *et al.*, 2018), and our work further supports this general observation. Though we show that RanBP2 and G3BP2 interact by coimmunoprecipitation (Figure 2F), we do not observe any detectable colocalization of either stress granules or P-bodies with cytoplasmic foci of RanBP2 (Supplemental Figure S7, A and B), which are likely annulate lamellae, but may also consist of membrane-free condensates (Palazzo *et al.*, 2022).

Finally, it is worth pointing out that dominant mutations in RanBP2 are associated with Acute Necrotizing Encephalopathy, a rare pediatric disease where otherwise normal individuals develop a cytokine storm upon infection by respiratory viruses (Neilson *et al.*, 2009; Jiang *et al.*, 2022). Indeed, RanBP2-dependent SUMOylation seems to regulate several branches of the innate immune response, including STAT1 signaling (Li *et al.*, 2024) and the production of Interleukin 6 and TNFα (Shen *et al.*, 2021), two key cytokines secreted during viral infection. Interestingly, RNA viruses sometimes trigger, and other times inhibit, stress granule assembly (Guan *et al.*, 2023). Thus, by modulating stress granule dynamics, RanBP2 may further modulate the activity of the innate immune response to viral infection. This may be taken advantage of by certain viruses. In *Drosophila* S2 cells, Cricket paralysis virus promotes the destruction of RanBP2, and this blocks stress granule formation in order to promote infection (Sadasivan *et al.*, 2022). Future work will need to be performed to determine whether mammalian viruses target stress granule formation by modulating RanBP2.

## MATERIALS AND METHODS

Request a protocol through *Bio-protocol*

### Cell culture, cell transfection, and sodium arsenite treatment

Human osteosarcoma cells (U2OS) were cultured in DMEM (Wisent) supplemented with 10% FBS (Wisent) and 1% penicillin–streptomycin (Wisent) at 37°C in a 5% CO_2_-humidified incubator. RanBP2-dE3 U2OS cells that lack RanBP2-mediated SUMOylation were generated previously by CRISPR-Cas9-mediated gene editing (Shen *et al.*, 2021). G3BP1–G3BP2 double deletion (ΔΔG3BP1/2) U2OS cells were a gift from the Anderson lab (Kedersha *et al.*, 2016). For transfection experiments, cells were plated 24 h before transfection and transfected at a confluency of 80 to 90% using JetPRIME (PolyPlus), following the manufacturer's protocol. For stress granule dynamics experiments, cells were treated with sodium arsenite (100 µM, Sigma-Aldrich) for up to 60 min, and recovered in normal media for up to 120 min.

### Plasmid constructs

The human *His6-SUMO1* (in pEFIRES), *His6-SUMO3* (in pcDNA3), and V5-Ubc9 plasmids were gifts from L. Frappier (De La Cruz-Herrera *et al.*, 2018). *pSUMO3* plasmid was a gift from Primo Schaer (Addgene plasmid # 52260) (Weber *et al.*, 2014). *pENTR4_G3BP2* was a gift from Thomas Tuschl (Addgene plasmid # 127105) (Meyer *et al.*, 2020). To generate the *G3BP2-HA-pcDNA3* construct, the G3BP2 ORF was amplified from *pENTR4_G3BP2* with forward primer: 5ʹ-GGTACCATGGTTATGGAGAAGCCCAGT-3ʹ and reverse primer: 5ʹ- CTCGAGTCAGCGACGCTGTCCTGTGAA-3ʹ, and subcloned into *pcDNA3.1* using KpnI and XhoI (New England Biolabs). The HA tag was then inserted at the 3ʹ end by restriction-enzyme–free cloning. The *G3BP2-K281R-HA* mutant was generated by site-directed mutagenesis with forward primer: 5ʹ-AGACCAGAAGTTCAATCTCAGCCACC-3ʹ, and reverse primer: 5ʹ-AGCTTCGACTCTTGGCTGTG-3ʹ. To generate the *FLAG-G3BP2-pET* construct, the G3BP2 ORF was first amplified from *G3BP2-HA-pcDNA3* with forward primer: 5ʹ-GATTGGTGGTATGGTTATGGAGAAGCCCAGTC-3ʹ and reverse primer: 5ʹ-AAGCTTGTCTTCAGCGACGCTGTCCTGTG-3ʹ, and then inserted into PCR-amplified *pET-SUMO* expression vector (forward primer: 5′-GCGTCGCTGAAGACAAGCTTAGGTATTTATTCGG-3′ and reverse primer: 5′-CCATAACCATACCACCAATCTGCTCACG-3′) using Gibson Assembly Master Mix (New England Biolabs). The His6-SUMO tag at the N-terminus of G3BP2 was then replaced with FLAG and a Gly-Ser linker by restriction-enzyme-free cloning. *pGEX* and *pGEX-RanBP2ΔFG* (aa 2553-2838) plasmids were gifts from F. Melchior (Pichler *et al.*, 2002). To change the origin of replication from ColE1 to p15A in the *pGEX* plasmids, the p15A sequence was first amplified from *pACYCDuet* (a gift from T. Moraes) using forward primer: 5ʹ-GATCAAAGGATCTTCTTGAGATCGTTTTG-3ʹ and reverse primer: 5ʹ-CGCGTTGCTGGCGTTTTTCCATAGGCTCC-3ʹ, and then inserted into PCR-amplified *pGEX* and *pGEX-RanBP2ΔFG* (forward primer: 5ʹ-AACGCCAGCAACGCGGCC-3ʹ and reverse primer 5ʹ-GAAGATCCTTTGATCTTTTCTACGGGGTCT-3ʹ) using In-Fusion Snap Assembly Master Mix (Takara Bio).

### Immunofluorescence

Cells on coverslips were fixed with 4% paraformaldehyde (Electron Microscopy Sciences) and permeabilized with 0.1% Triton X-100 Surfact-Amps (Thermo Fisher Scientific). For SUMO immunostaining, cells were fixed with ice-cold 100% methanol and rehydrated in PBS. Subsequently, the coverslips were incubated with primary antibodies against RanBP2/Nup358 (a kind gift from R. Wozniak and E. Coutavas [Wu *et al.*, 1995], 1:750), G3BP1/2 (Abcam, ab56574, 1:1000), Dcp1a (Sigma, WH0053802M6, 1:100), HA (Sigma, H6908 or H3663, 1:1000), TIA1 (Invitrogen, MA5-26474, 1:200 or Santa Cruz, sc-1751, 1:100), Caprin (Proteintech, 15112-1-AP, 1:200), PABP (Abcam, ab21060, 1:200), SUMO1 (Proteintech, 10329-1-AP, 1:300), SUMO2/3 (Proteintech, 11251-1-AP, 1:300), and Alexa488-conjugated anti-mouse IgG (Invitrogen, A-21202, 1:200) or anti-rabbit IgG (Invitrogen, A-21206, 1:200), Alexa546-conjugated anti-mouse IgG (Invitrogen, A-10036, 1:200), Alexa647-conjugated anti-mouse IgG (Invitrogen, A-31571, 1:200) or anti-rabbit IgG (Invitrogen, A-21244, 1:200) or anti-goat IgG (Invitrogen, A-21447, 1:200) secondary antibodies. Finally, the coverslips were mounted onto glass slides using DAPI Fluoromount-G stain mounting solution (Southern Biotech).

### Imaging and image analysis

Coverslips were imaged with a Ti-E inverted fluorescence Nikon microscope using a 60X phase 2 oil objective and a Coolsnap HQ2 14-bit CCD camera, controlled using Nikon Imaging Software (NIS) Elements. For stress granule dynamic analysis, the percentage of cells with stress granules, the number of stress granules per cell, and the percentage area of the cytoplasm occupied by stress granules were quantified using CellProfiler (Carpenter *et al.*, 2006). Nuclei were identified with the Minimum Cross-Entropy thresholding method. Cell outlines were either identified by the Distance method from the nuclear signal for staining of endogenous G3BP1/2, or by the Watershed method for the HA staining of transfected G3BP2-HA. Stress granules were identified by either the Sauvola or Otsu thresholding methods. For each experiment, at least 40 cells were analyzed in each condition. For P-body analysis, the number of P-bodies per cell was counted manually, with at least 25 cells analyzed per condition in each experiment. For stress granule–P-body colocalization analysis, the number of undocked stress granules or P-body (not in contact with one another), docked stress granule–P-body (in direct contact with each other or partially overlap), and colocalized stress granule–P-body (fully overlap or one encompassing the other) in each cell was counted manually. For each experiment, at least 20 cells were analyzed in each condition. For G3BP1/2 and SUMO2/3 colocalization and intensity analyses, the NIS Elements Advanced Research Analysis software was used, and quantification was performed on raw, unprocessed images. Rectangular regions of interest were first drawn to encompass individual G3BP1/2-positive cytoplasmic foci. Pearson correlation coefficients between G3BP1/2 and SUMO2/3 signals were calculated by the NIS analysis software. The area and the mean SUMO2/3 integrated fluorescence intensity within each region of interest were also recorded. Background intensity was defined as the average SUMO2/3 intensity in the cytoplasmic region lacking visible G3BP1/2 foci. SUMO2/3 intensity for each region of interest was then calculated by subtracting background intensity from the mean intensity and multiplying by the area. For each experiment, at least 200 foci were analyzed in each condition. Images shown in figures were adjusted for brightness and contrast using Photoshop (Adobe).

### In vivo SUMOylation assay

In vivo G3BP2 SUMOylation was analyzed in unmodified and RanBP2-dE3 U2OS cells as described previously (Shen *et al.*, 2021). Briefly, the cells were transfected with plasmids expressing His6-SUMO1 or His6-SUMO3, V5-Ubc9 and/or G3BP2-HA or empty plasmid as negative control using JetPRIME reagent (PolyPlus) according to the manufacturer's instructions. After transfection and/or sodium arsenite treatment, the cells were harvested, and 10% were collected as input and analyzed directly by immunoblotting. The remaining 90% of the cells were resuspended in lysis buffer containing 6 M Guanidinium-HCl, 100 mM K_2_HPO_4_, 20 mM Tris-HCl (pH 8.0), 100 mM NaCl, 0.1% Triton X-100, and 10 mM imidazole, and incubated on ice for 30 min. Lysates were passed through 26-gauge needles five times and cleared by centrifugation at 16,000 × g for 30 min at 4°C. Purification of the His6-SUMO1- or His6-SUMO3-conjugates was performed with 50 µL of Ni^2+^-NTA agarose beads (Qiagen) prewashed with lysis buffer, and incubated for 3 h at room temperature with end-over-end rotation. The beads were washed once with 1 mL lysis buffer, and three times with 1 mL wash buffer containing 8 M urea, 0.1 M Na_2_HPO_4_/NaH_2_PO_4_ (pH 6.4), 10 mM Tris-HCl (pH 6.4), 10 mM imidazole, 10 mM β-mercaptoethanol, and 0.1% Triton X-100 before elution in 2X Laemmli sample buffer. For SUMOylation assays with His6-SUMO1, the stringency of the wash buffer was increased by using 1% Triton X-100 and 20 mM imidazole.

### *E. coli* BL21-based SUMOylation assay

The *pSUMO3* plasmid (which coexpresses human His6-SUMO3, SUMO E1 enzymes SAE1/SAE1 and SUMO E2 enzyme Ubc9) and the *FLAG-G3BP2-pET* plasmid were transformed individually (as controls) or together into BL21 (DE3) *E. coli* cells (Thermo Fisher Scientific). Cells were then made chemically competent again using calcium chloride and transformed with either *pGEX* (expresses GST control) or *pGEX-RanBP2ΔFG* (expresses GST-tagged RanBP2 E3 domain fragment) plasmids with p15A origin of replication. Clones coexpressing two or three plasmids were verified by restriction enzyme digestion. Overnight precultures were diluted 1:25 into fresh LB medium and grown at 37°C to an OD_600_ of ∼0.7 to 0.8. Cultures were maintained under selective pressures using 50 mg/L of ampicillin, kanamycin, or streptomycin for single plasmid expression, half the concentration of each antibiotic for co-expression of two plasmids, or one-third the concentration for coexpression of three plasmids. Protein expression was induced with 0.01 mM IPTG at 16°C for 4 h. Cells were harvested by centrifugation, and 1% was collected as input and directly analyzed by immunoblotting. The remaining cells were lysed in lysis buffer containing 6 M Guanidinium-HCl, 100 mM K_2_HPO_4_, 20 mM Tris-HCl (pH 8.0), 100 mM NaCl, 0.1% Triton X-100, and 10 mM imidazole, and incubated at room temperature with rotation for 1 h. Lysates were cleared by centrifugation at 10,000 × g for 30 min at 4°C. Purification of His6-SUMO3-conjugates was performed with 100 µL of Ni^2+^-NTA agarose beads (Qiagen) prewashed with lysis buffer, and incubated overnight with rotation at 4°C. The beads were washed once with 1 mL lysis buffer, and three times with 1mL wash buffer containing 8 M urea, 0.1 M Na_2_HPO_4_/NaH_2_PO_4_ (pH 6.4), 10 mM Tris-HCl (pH 6.4), 10 mM imidazole, 10 mM β-mercaptoethanol, and 0.1% Triton X-100 before elution in 2X Laemmli sample buffer.

### Immunoprecipitation

Whole-cell extracts were collected from unmodified and RanBP2-dE3 U2OS cells and lysed in lysis buffer containing 50 mM Tris-HCl (pH8.0), 150 mM NaCl, 1% NP-40, cOmplete EDTA-free Protease Inhibitor Cocktail (Roche), and 10 mM PMSF at 4°C with end-over-end rotation for 30 min. The lysates were passed through 26-gauge needles five to 10 times, and cleared by centrifugation at 16,000 × g for 40 min at 4°C. For the supernatant, 10% was collected as input and analyzed directly by immunoblotting, and the remaining was incubated with Protein A Sepharose beads (Thermo Fisher Scientific) coupled to rabbit IgG (Santa Cruz Biotechnology, sc-2027) or anti-G3BP2 (Proteintech, 16276-1-AP, 4 µg per IP) overnight with rotation at 4°C. The beads were washed three times with lysis buffer, and bound proteins were eluted by heating at 95°C for 10 min in 2X Laemmli sample buffer.

### Immunoblotting

Cells were lysed with 2X Laemmli sample buffer and boiled for 10 min. Samples were separated by SDS–PAGE gels and transferred onto nitrocellulose membranes for immunoblotting. The membranes were incubated with primary antibodies against RanBP2/Nup358 (a kind gift from R. Wozniak and E. Coutavas, [Wu *et al.*, 1995], 1:10,000), RanGAP1 (Santa Cruz Biotechnology, sc-28322, 1:750), HA (Sigma, H6908, 1:1000 dilution), G3BP2 (Proteintech, 16276-1-AP, 1:1000), His (Abcam, ab18184, 1:1000), SUMO1 (Proteintech, 10329-1-AP, 1:2000), SUMO2/3 (Proteintech, 11251-1-AP, 1:2000), GST (Cell Signaling Technology, 2624T, 1:1000), Tubulin (Sigma, 05-829, 1:1000), GAPDH (Cell Signaling Technology, 2188S, 1:1000), and Vinculin (Abcam, ab129002, 1:5000) and subsequently with the relevant horse radish peroxidase (HRP)–conjugated anti-rabbit or anti-mouse (Cell Signaling Technology, 7074S or 7076S, 1:2000) secondary antibodies. Immobilon Crescendo Western HRP substrate (Millipore) and the Versadoc system (Bio-Rad) were used to visualize the blots. ImageJ (NIH) was used for densitometry analysis.

### Mass spectrometry analysis

Similar to the SUMOylation assay described above, except that unmodified and RanBP2-dE3 U2OS cells were transfected with a plasmid expressing His6-SUMO3, and lysed with lysis buffer containing 50 mM Tris-HCl (pH 8.0), 150 mM NaCl, 1% Triton X-100 with cOmplete, EDTA-free Protease Inhibitor Cocktail (Roche). His6-SUMO3-conjugates were then purified on a nickel column and eluted for mass spectrometry analysis.

### Sequence alignment

Protein sequences were obtained from Ensembl Release 103 (Howe *et al.*, 2021), aligned with the Clustal Omega Multiple Sequence Alignment tool (Sievers *et al.*, 2011), and edited with BioEdit (Hall, 1999).

### Statistical analysis

Statistical tests were performed using Student's *t* test to calculate *p*-values. A *p*-value of ≤0.05 was considered as statistically significant. Results of statistical analysis are included in the corresponding figures.

## Supporting information





## Abbreviations used:

Dcp1a: decapping enzyme 1a
FMR1: fragile X messenger ribonucleoprotein 1
FUS: fused in sarcoma
G3BP: ras GTPase-activation protein-binding protein
PABP: poly(A) binding protein P-body processing body
RanBP2: ran-binding protein 2
RNF4: RING finger protein 4
RNP: ribonucleoprotein
StUbL: SUMO-targeted ubiquitin ligase
SUMO: small ubiquitin-like modifier
TDP-43: transactive response DNA-binding protein 43
TIA1: T-cell intracellular antigen 1
TRIM28: tripartite motif containing 28.
