# Supplementary material for: RanBP2-dependent SUMOylation of G3BP2 inhibits formation, and promotes disassembly, of stress granules
Source: Mol Biol Cell. 2026 Jun 10;37(7):ar69. doi: 10.1091/mbc.E25-03-0135 (PMC13329889; doi:10.1091/mbc.E25-03-0135)
Supplement: Supplementary file 1 [file mbc-37-ar69-s001.pdf]

Supplemental Materials

Molecular Biology of the Cell

Wang *et al*.

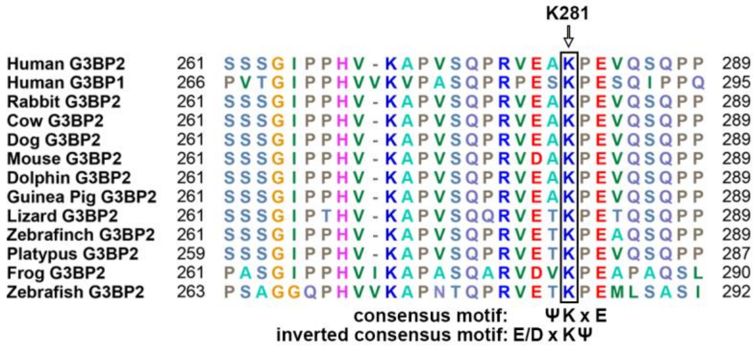

Supplemental Figure 1. Conservation of the region surrounding lysine 281 of G3BP2.

G3BP2 protein sequences from various organisms and human G3BP1 sequence were aligned using the Clustal Omega Multiple Alignment tool. The region surrounding lysine 281 is shown. Consensus and inverted consensus SUMOylation motifs were shown at the bottom as reference, where ψ represents hydrophobic amino acids and x represents any amino acid.

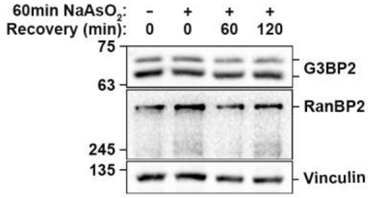

Supplemental Figure 2. Arsenite stress and recovery do not affect the levels of endogenous G3BP2.

U2OS cells were treated with 100µM sodium arsenite for 60min, and allowed to recover in arsenite-free media for up to 120min. At various time points, cells were harvested and analyzed by immunoblotting for the indicated proteins.

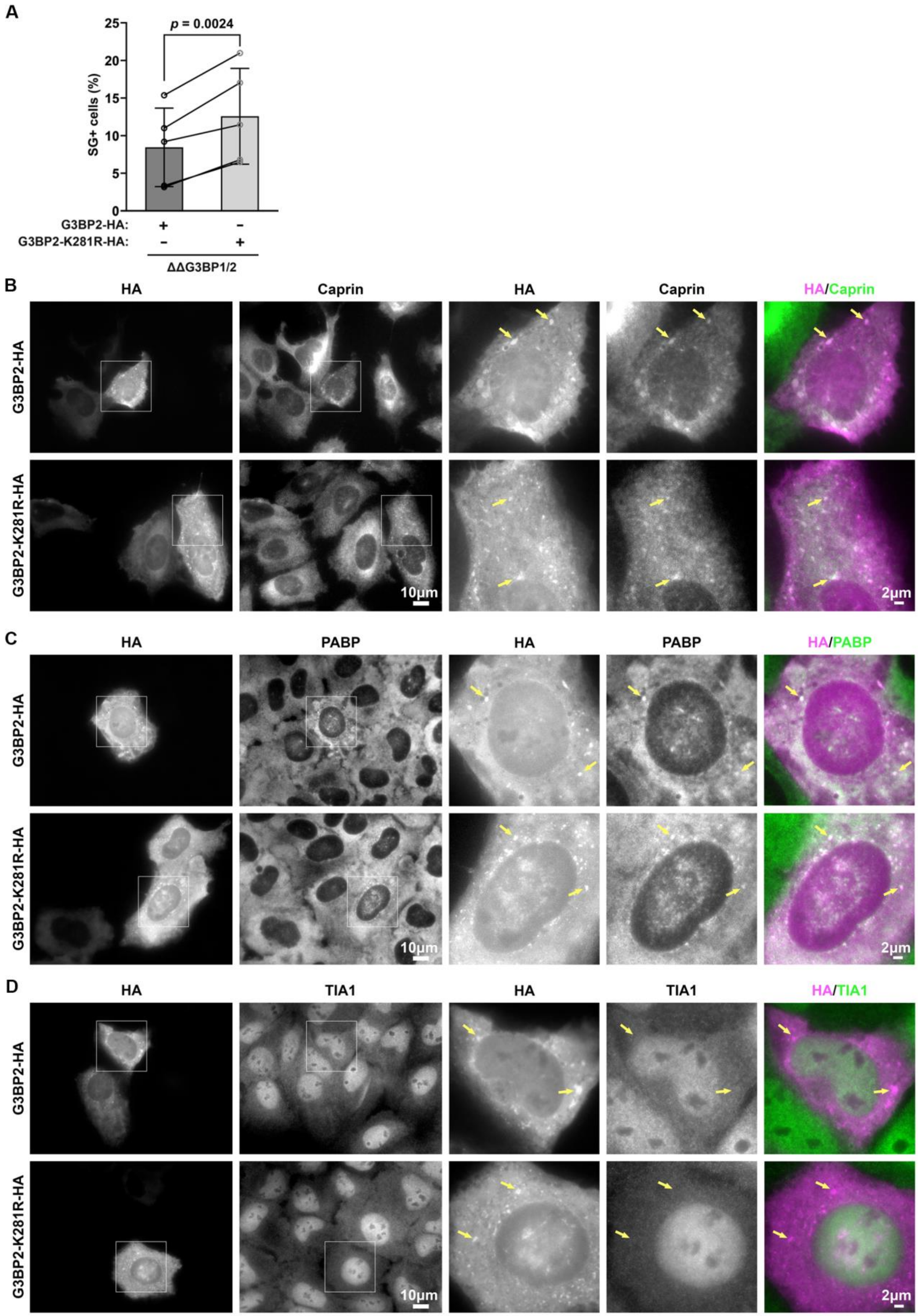

A
p = 0.0024
SG+ cells (%)
25
20
15
10
5
0
G3BP2-HA:
G3BP2-K281R-HA:
ΔΔG3BP1/2
B
HA
Caprin
HA/Caprin
G3BP2-HA
G3BP2-K281R-HA
10µm
2µm
C
PABP
HA/PABP
D
TIA1
HA/TIA1

Supplemental Figure 3. SUMOylation-deficient G3BP2 has a higher propensity to trigger stress granules assembly.

A) ΔΔG3BP1/2 U2OS cells were transfected with *G3BP2-HA* or *G3BP2-K281R-HA* for 24hr, fixed and immunostained for HA to identify transfected cells and use as a stress granule marker. Quantification of the percentage cells displaying stress granules is shown. Each bar represents the averages ± SEM of five independent experiments, and the data points from each experiment are shown in pairings.

B-D) ΔΔG3BP1/2 U2OS cells were transfected with *G3BP2-HA* or *G3BP2-K281R-HA* for 24hr, fixed and immunostained for HA to identify transfected cells and use as a stress granule marker, and Caprin (B), PABP (C) or TIA1 (D) as another markers for stress granules. Each row represents a single field of view. The areas for the insets are indicated with white boxes and representative stress granules are highlighted with yellow arrows. Scale bar: whole cell = 10µm, insets = 2µm.

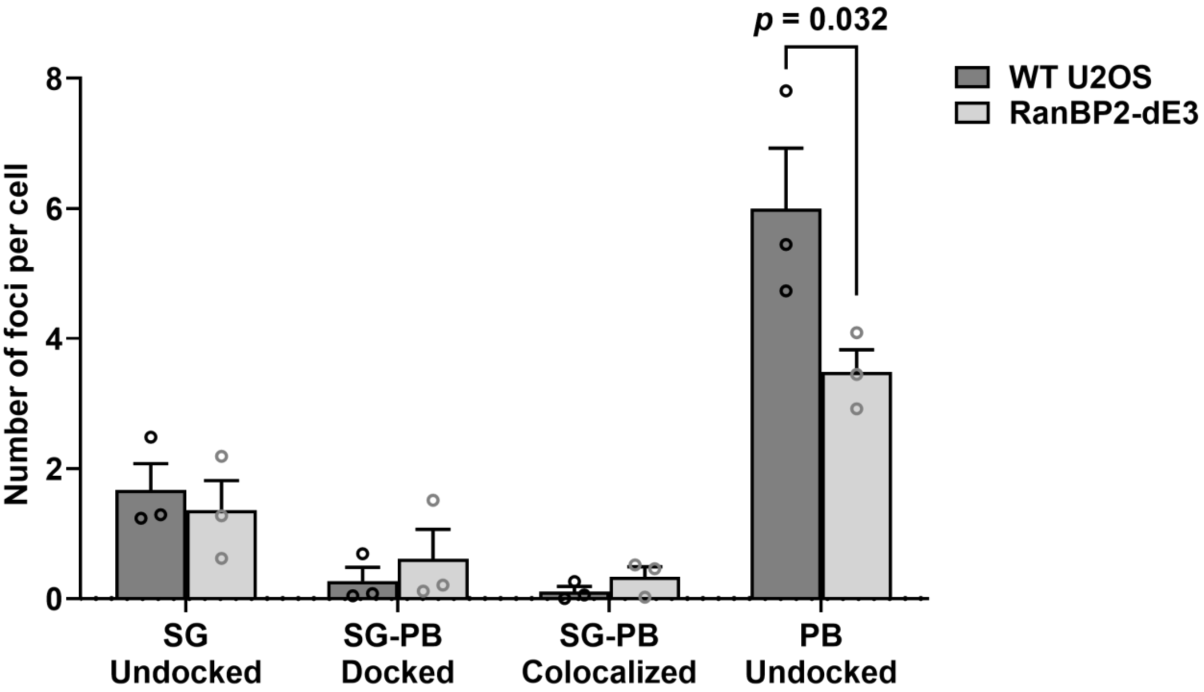

Supplemental Figure 4. The docking and colocalization events of stress granules and P-bodies are transient.

Unmodified (WT) and RanBP2-dE3 U2OS cells were treated with 100µM sodium arsenite for 60min and allowed to recover in arsenite-free media for 60min. Cells were fixed in ice-cold methanol and immunostained for Dcp1a as a P-body marker and TIA1 as a stress granule marker. The number of undocked stress granule or P-body (not in contact with one another), docked stress granule-P-body (in direct contact with each other or partially overlap) and colocalized stress granule-P-body (fully overlap or one encompassing the other) in each cell were quantified. Each bar represents the averages ± SEM of three independent experiments.

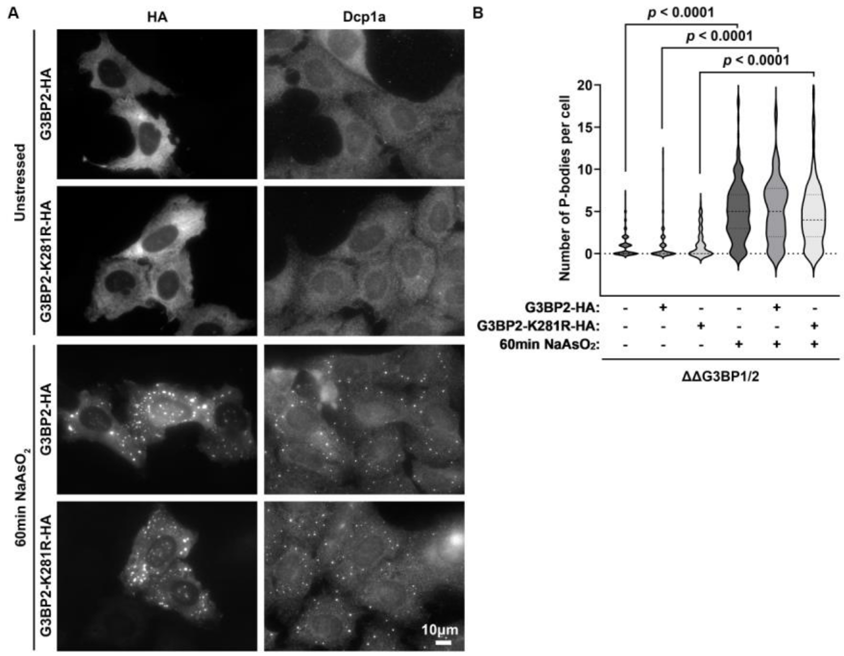

Supplemental Figure 5. P-body formation in ΔΔG3BP1/2 expressing G3BP2-HA and G3BP2-K281R-HA before and after arsenite stress.

A) ΔΔG3BP1/2 U2OS cells were transfected with *G3BP2-HA* or *G3BP2-K281R-HA* for 24hr and treated with 100µM sodium arsenite for 60min. Cells were fixed and immunostained for HA to identify transfected cells and use as a stress granule marker, and Dcp1a as a P-body marker. Each row represents a single field of view. Scale bar = 10µm.

B) For images in (A), the number of P-bodies per cell were quantified manually. At least 40 cells were quantified per condition across two independent experiments.

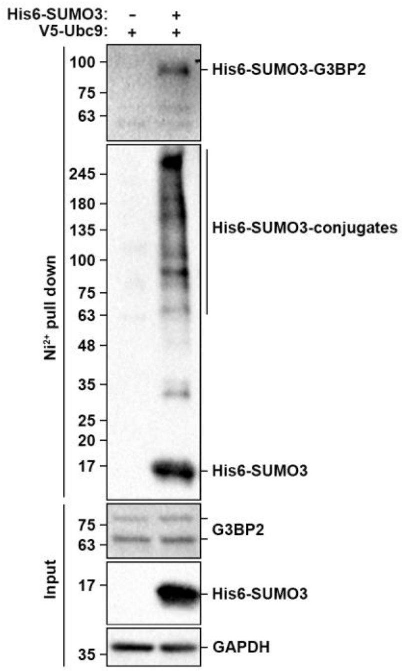

Supplemental Figure 6. SUMOylation of endogenous G3BP2.

Unmodified (WT) U2OS cells were transfected with *His6-SUMO3 and SV5-Ubc9* as indicated. 24hr post transfection, the transfected cells were lysed under denaturing conditions, and proteins covalently attached to His6-SUMO3 were isolated by nickel column (Ni^{2+} pull down), and analyzed by immunoblotting with anti-G3BP2 for endogenous G3BP2 and anti-His for free and total His6-SUMO3-conjugates. Cell lysates were also directly analyzed as Input for the indicated proteins.

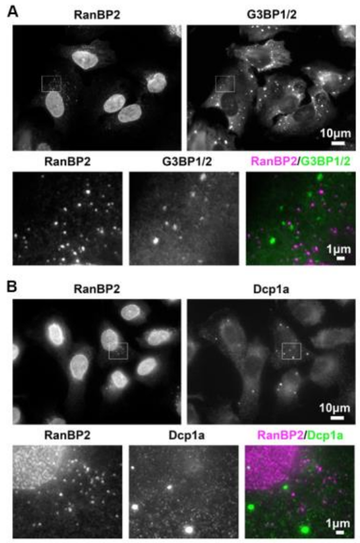

Supplemental Figure 7. Cytoplasmic foci of RanBP2 do not co-localize with either stress granules or P-bodies.

U2OS cells were immunostained for RanBP2 and G3BP1/2 (stress granule marker) after treatment with 100µM sodium arsenite for 60min (A), or for RanBP2 and Dcp1a (P-body marker) under unstressed conditions (B). The areas for the insets are indicated with white boxes. Each row represents a single field of view. Scale bar: whole cell = 10µm, insets = 1µm.
