# Supplementary material for: RanBP2-dependent SUMOylation of G3BP2 inhibits formation, and promotes disassembly, of stress granules
Source: Mol Biol Cell. 2026 Jun 10;37(7):ar69. doi: 10.1091/mbc.E25-03-0135 (PMC13329889; doi:10.1091/mbc.E25-03-0135)
Supplement: Supplementary file 2 [file reviewer_reportE25-03-0135.pdf]

RanBP2-dependent SUMOylation of G3BP2 inhibits formation, and promotes disassembly, of stress granules

Yifan Wang, Javid Guliyev, Sean Ihn, Gaelen Moore, Fermisk Saleh, Hyun Lee, and Alexander Palazzo

Corresponding author(s): Alexander Palazzo, University of Toronto

Review Timeline:

Submission Date:	2025-03-26
Editorial Decision:	2025-04-27
Revision Received:	2026-04-23
Accepted:	2026-05-14

Editor-in-Chief: John Aitchison

Transaction Report:

(Note: With the exception of the correction of typographical or spelling errors that could be a source of ambiguity, letters and reports are not edited. The original formatting of letters and referee reports may not be reflected in this compilation.)

RE: Manuscript #E25-03-0135
TITLE: RanBP2-dependent SUMOylation of G3BP2 inhibits formation, and promotes disassembly, of stress granules

Dear Alex,

Thank you for submitting your paper (#E25-03-0135) entitled "RanBP2-dependent SUMOylation of G3BP2 inhibits formation, and promotes disassembly, of stress granules". Two experts in the field have now evaluated your manuscript. Both Reviewers have raised various concerns that I ask you address in a revised version of your manuscript. Among their points, both Reviewers have requested further evidence that would support a direct role for RanBP2 in the SUMOylation of G3PB2. Reviewer 2 also has more general concerns regarding the lack of a clear rationale for your analysis of both stress granules and p-bodies and how their mechanisms of regulation may be jointly regulated by SUMOylation. Once you have addressed these and other stated concerns, please provide a point-by-point outline of your responses to the Reviewers' comments and indicate how you have incorporated these into a revised manuscript.

Best regards,

Rick

Richard Wozniak
Monitoring Editor
Molecular Biology of the Cell

Dear Dr. Palazzo,

The review of your manuscript, referenced above, is now complete. The Monitoring Editor has decided that your manuscript is not acceptable for publication at this time, but may be deemed acceptable after specific revisions are made, as described in the Monitoring Editor's decision letter above and the reviewer comments below.

A reminder: Please do not contact the Monitoring Editor directly regarding your manuscript. If you have any questions regarding the review process or the decision, please contact the MBoC Editorial Office (mboc@ascb.org).

When submitting your revision include a rebuttal letter that details, point-by-point, how the Monitoring Editor's and reviewers' comments have been addressed. (The file type for this letter must be "rebuttal letter"; do not include your response to the Monitoring Editor and reviewers in a "cover letter.") Please bear in mind that your rebuttal letter will be published with your paper if it is accepted, unless you have opted out of publishing the review history.

Authors are allowed 90 days to submit a revision. If this time period is inadequate, please contact us at mboc@ascb.org.

Revised manuscripts are assigned to the original Monitoring Editor whenever possible. However, special circumstances may preclude this. Also, revised manuscripts are often sent out for re-review, usually to the original reviewers when possible. The Monitoring Editor may solicit additional reviews if it is deemed necessary to render a completely informed decision.

In preparing your revised manuscript, please follow the instruction in the Information for Authors (www.molbiolcell.org/info-for-authors). In particular, to prepare for the possible acceptance of your revised manuscript, submit final, publication-quality figures with your revision as described.

To submit the rebuttal letter, revised manuscript, and figures, use this link: Link Not Available

MBoC offers the option to publish your work with immediate open access. Open access can increase the discoverability and usability of your research. If you would like to publish your paper with immediate open access but did not select this option during initial submission, please contact the editorial office at mbc@ascb.org.

Authors of Articles and Brief Communications are also encouraged to create a short video abstract to accompany their article when it is published. These video abstracts, known as Science Sketches, are up to 2 minutes long and will be published on YouTube and then embedded in the article abstract. Science Sketch Editors on the MBoC Editorial Board will guide you as you prepare your video. Information about how to prepare and submit a video abstract is available at www.molbiolcell.org/science-sketches. Please contact mboc@ascb.org if you are interested in creating a Science Sketch.

Sign up for MBoC's new content alerts here https://tinyurl.com/3bcsyk3e

Thank you for submitting your manuscript to MBoC. We look forward to receiving your revised paper.

Sincerely,

Eric Baker
Journal Production Manager
MBoC Editorial Office
mbc@ascb.org

Reviewer #1 (Remarks to the Author):

The work by Wand et al reports that the SUMOylation of the stress granule component, G3BP2, is dependent on Ranbp2. The absence of Ranbp2-dependent SUMOylation activity hastens the assembly of stress granules, whereas it slows their disassembly by oxidative stress and after recovery from this insult (sodium arsenite). K281 of G3BP2 appears to undergo SUMOylation by Ranbp2. In general, the observations and data presented appear to support the claims made by the authors, but this study also raises several questions that need to be addressed as pointed out hereunder.

1. Typically, E3-ligases (e.g., Ranbp2) provide substrate selectivity for E2-ligases (e.g., Ubc9). This implies that a ternary complex is formed between Ranbp2, Ubc9, and G3BP2. However, these are shown neither by co-immunoprecipitation assays nor by co-immunolocalization (e.g., in stress granules) in cultured cells. The authors should specifically comment whether experiments have been done to this effect and their outcomes.

2. The major weakness of this study is that it relies on the over-expression of substrates, Ubc9, or SUMO to draw the supported claims. Potential limitations of this approach alone need to be discussed. For example, in Fig. 3D the authors claim the need to overexpress Ubc9 because SUMO-conjugates are short-lived/labile. Although true, it seems that this issue is amplified because the authors apparently do not use N-ethylmaleimide (NEM) when preparing cell lysates. It is well known that NEM inhibits deSUMOylating enzymes (e.g., SENPs). Further, SUMOylated RanGAP may be a useful surrogate marker for the status of isopeptide bonds between SUMO and target proteins. These issues need to be addressed in the context of the data presented.

3. It is unclear why G3BP2 is conjugated with SUMO-2 instead of SUMO-1. If G3BP1 does not present a similar SUMOylation motif as G3BP2, what is the SUMOylation status of the former by Ranbp2 and oxidative stress? G3BP1 may serve as a good internal control (including in co-IP assays).

4. In Fig. 3D, G3BP2 shows up as a doublet. In the discussion, it is claimed that these are splicing products. However, His6-SUMO2-G3BP2 shows as a singlet. Are the authors claiming that only one isoform undergoes SUMOylation? Why?

5. SUMOylation of a substrate should cause partial depletion of unconjugated substrate, whereas the opposite should also be true (e.g., SUMOylation of RanGAP, Fig. 1A). However, this does not appear to be clear from data presented in Figs. 3B and 3D. In fact, Fig. 3D appears to show depletions of both conjugated and unconjugated G3BP2 in the recovery phase.

6. What are the immunoreactivities of stress granules for SUMO1/2 in the cells used?

Minor points: Abbreviations of G3BP1/2 need to be described in full.

Reviewer #2 (Remarks to the Author):

This report analyzes the relationship between RanBP2-dependent SUMOylation of G3BP2 and stress granule (SG) dynamics. The authors monitored SG formation and disassembly after sodium arsenite treatment and wash out in mutant U2OS cells that lack the SUMO ligase domain of RanBP2 (RanBP2-de3 cells). RanBP2-de3 cells show accelerated assembly of SGs upon sodium arsenite treatment and these SG persist longer than in control cells, although the peak number of SG was lower than controls (Figure 1). It has previously been demonstrated that RanBP2 depletion decreases p-body assembly; the authors show that RanBP2-de3 cells have fewer p-bodies, indicating that the steady-state level of p-bodies can be impacted by the mutation of RanBP2. Mass spec analysis of Ni2+ pull-down fractions from His6-SUMO2-expressing cells shows decreased levels of SG components G3BP1 and G3BP2 in the RanBP2-de3 cells compared to control U2OS cells, suggesting that these proteins may be SUMOylated in a RanBP2-dependent manner. The authors show that mutation of G3BP2 (K281R) decreases its SUMOylation levels, indicating this residue as a SUMO2 conjugation site. They further show that G3BP2 SUMOylation decreases with sodium arsenite treatment of control U2OS cells and then decreases further during the recovery period. Finally, the authors utilize U2OS cells lacking both G3BP1 and G3BP2 (G3BP1/2) to express either G3BP2-HA or G3BP2-K281R-HA

to measure their relative capacity for SG assembly. The authors observed that cells expressing G3BP2-K281R-HA had higher persistent SG levels in unstressed conditions, as well as modestly accelerated assembly of SGs after sodium arsenite treatment and SG that persisted longer than in G3BP2-HA-expressing cells during washout.

Taken together, these data suggest that while SUMOylation of K281 on G3BP2 by RanBP2 is not essential for SG formation or disassembly, it may contribute to the regulation of these processes. As the authors note, it is counterintuitive that SUMOylation levels of G3BP2 are not stimulated by stress, but rather decrease upon stress and fall further during recovery (Figure 3E), so that there is not a simple correlation between G3BP2 SUMOylation and the level of SG assembly. This manuscript would be far stronger if the authors could provide the logic for this observation and biochemical evidence of the regulatory mechanism underlying these phenomena. The manuscript could also be strengthened with a more substantial link between their observations on SG and p-bodies, which currently seems obscure.

Additional points to address:

• The conclusion that RanBP2 mediates SUMOylation of G3BP2 at K281R would be substantially strengthened by in vitro assays demonstrating SUMOylation with purified proteins to show the direct enzyme-substrate relationship. (Werner et al., 2009. PMID: 19107418)

• In Figure 2, the author show that the number of p-bodies is lower in RanBP2-de3 cells, leading them to conclude that "the effects previously reported are likely due to changes in SUMOylation by RanBP2" (p.6). While this suggestion is consistent with the data, it is an over-interpretation. The RanBP2 mutations in RanBP2-de3 cells impact other aspects of RanBP2 function. For example, these mutations eliminate the RanGAP1 binding site at the nuclear pore, rendering it cytoplasmic and susceptible to de-SUMOylation (Zhu et al. 2009. PMID: 19285941). They also decrease the overall level of RanBP2 (Figure 1A,B). The authors should change the text to suitably acknowledge this ambiguity.

• Even if RanBP2 regulates p-body assembly primarily through SUMOylation, the findings in this manuscript seem preliminary because the targets may be unrelated to those involved in SG control. It is not clear how these findings coherently fit into the SG regulatory mechanism, but the manuscript would be strengthened if the logic of this link were clarified.

• The third column of the table in Figure 3A is not a ratio of the first two columns. It's not clear what it represents, so please clarify in the figure legend.

• Figure 3D,E shows that G3BP2 SUMOylation decreases with sodium arsenite treatment of control U2OS cells and then decreases further during the recovery period. Is this pattern altered in RanBP2-de3 cells, which still show G3BP2 SUMOylation at roughly half the level of untreated U2OS controls (Figure 3C)?

Dear Rick,

Below we provide a point-by-point response to the reviewers' comments. We believe that we have answered most of their concerns. Our responses are in red.

Thanks,

Alex

Dear Alex,

Thank you for submitting your paper (#E25-03-0135) entitled "RanBP2-dependent SUMOylation of G3BP2 inhibits formation, and promotes disassembly, of stress granules". Two experts in the field have now evaluated your manuscript. Both Reviewers have raised various concerns that I ask you address in a revised version of your manuscript. Among their points, both Reviewers have requested further evidence that would support a direct role for RanBP2 in the SUMOylation of G3PB2.

We thank you and the reviewers for prompting us to test whether RanBP2 directly SUMOylates G3BP2. Using a bacterial recombination system, we now show that the RanBP2 E3 domain can promote SUMOylation of G3BP2 (Figure 2G).

Reviewer 2 also has more general concerns regarding the lack of a clear rationale for your analysis of both stress granules and p-bodies and how their mechanisms of regulation may be jointly regulated by SUMOylation.

Again, we thank you and reviewer 2 for prompting us to more closely examine the relationship between stress granules and P-bodies. We now show that in the absence of RanBP2-dependent SUMOylation, a subset of P-bodies merge with stress granules in arsenite-treated cells (Figure 5C-F).

Once you have addressed these and other stated concerns, please provide a point-by-point outline of your responses to the Reviewers' comments and indicate how you have incorporated these into a revised manuscript.

Again, we thank you for your time and patience. Beyond the two critical experiments described above, we have included a number of new pieces of data, which includes:

- **the co-immunoprecipitation of endogenous G3BP2 with endogenous RanBP2 in an E3-dependent manner (Figure 2F)**
- **the localization of SUMO2/3, but not SUMO1, to stress granules and how this is altered in RanBP2-dE3 cells (Figure 3B)**
- **a re-examination of G3BP2-SUMOylation kinetics without the over-expression of Ubc9 (Figure 3E-F)**

We believe that we have addressed most of the concerns of the two reviewers.

Best regards,

Rick

Richard Wozniak
Monitoring Editor
Molecular Biology of the Cell

Reviewer #1 (Remarks to the Author):

The work by Wand et al reports that the SUMOylation of the stress granule component, G3BP2, is dependent on Ranbp2. The absence of Ranbp2-dependent SUMOylation activity hastens the assembly of stress granules, whereas it slows their disassembly by oxidative stress and after recovery from this insult (sodium arsenite). K281 of G3BP2 appears to undergo SUMOylation by Ranbp2. In general, the observations and data presented appear to support the claims made by the authors, but this study also raises several questions that need to be addressed as pointed out hereunder.

We thank the reviewer for their assessment. We now provide additional experiments that we believe addresses most of the reviewer's concerns.

1. Typically, E3-ligases (e.g., Ranbp2) provide substrate selectivity for E2-ligases (e.g., Ubc9). This implies that a ternary complex is formed between Ranbp2, Ubc9, and G3BP2. However, these are shown neither by co-immunoprecipitation assays nor by co-immunolocalization (e.g., in stress granules) in cultured cells. The authors should specifically comment whether experiments have been done to this effect and their outcomes.

We agree that providing some data of direct interaction would strengthen our manuscript. To this end, we now show that RanBP2 interacts with endogenous G3BP2 by co-immunoprecipitation experiments (Figure 2F) and that RanBP2 directly SUMOylates G3BP2 using a recombinant SUMOylation system in *E. coli* (Figure 2G).

2. The major weakness of this study is that it relies on the over-expression of substrates, Ubc9, or SUMO to draw the supported claims. Potential limitations of this approach alone need to be discussed. For example, in Fig. 3D the authors claim the need to overexpress Ubc9 because SUMO-conjugates are short-lived/labile. Although true, it seems that this issue is amplified because the authors apparently do not use N-ethylmaleimide (NEM) when preparing cell lysates. It is well known that NEM inhibits deSUMOylating enzymes (e.g., SENPs). Further, SUMOylated RanGAP may be a useful surrogate marker for the status of isopeptide bonds between SUMO and target proteins. These issues need to be addressed in the context of the data presented.

We thank the reviewer for their comments. We would like to point out that we performed the pull-down assays in denaturing buffer (6M guanidine-HCl) to inactivate all deSUMOylating enzymes upon cell lysis and to capture all proteins that are covalently conjugated to His6-SUMO. When we assess whether endogenous G3BP2 is SUMOylated

under these conditions in the absence of Ubc9, we consistently see a faint band (see figure below) that is SUMO3-dependent.

The faint nature of this band prompted us to perform the assay with exogenous G3BP2-HA to obtain a more robust signal that would be easier to analyse. Nevertheless, due to the fact that the mass spectrometry results were with endogenous G3BP2, without Ubc9 over-expression, we are confident that the modification does occur on endogenous protein and that this is dependent on RanBP2 (Figure 2A). In the newest version of the manuscript, we removed the Ubc9 over-expression experiments (although we do show one experiment in Supplemental Figure 6 as it provides some data about which isoforms of G3BP2 are SUMOylated, see discussion) and perform these assays with only G3BP2-HA over-expression (Figure 2B, 2D and 3E). We point out in the text that detecting SUMOylated proteins is tricky and typically done with over-expressed proteins.

3. It is unclear why G3BP2 is conjugated with SUMO-2 instead of SUMO-1.

We now show that G3BP2 can be modified by both SUMO1 and SUMO3 (Figure 2B-E). Furthermore, we now show that SUMO2/3, but not SUMO1, is enriched in stress granules and that this is partially dependent on RanBP2 (Figure 3A-D). Incidentally, we discovered that the His6-SUMO construct that we over-expressed is SUMO3 rather than SUMO2. We have thus corrected the text to reflect this. We should note that these two proteins differ only by 3 amino acids and are considered to have almost identical properties.

If G3BP1 does not present a similar SUMOylation motif as G3BP2, what is the SUMOylation status of the former by Ranbp2 and oxidative stress? G3BP1 may serve as a good internal control (including in co-IP assays).

Although we focus our main efforts on G3BP2, our mass spectrometry analysis detected G3BP1 SUMOylation, though it was less dependent on RanBP2 (Figure 2A). When we repeated the nickel pull down experiment with G3BP1-YFP, we failed to detect SUMOylated G3BP1 in our cells.

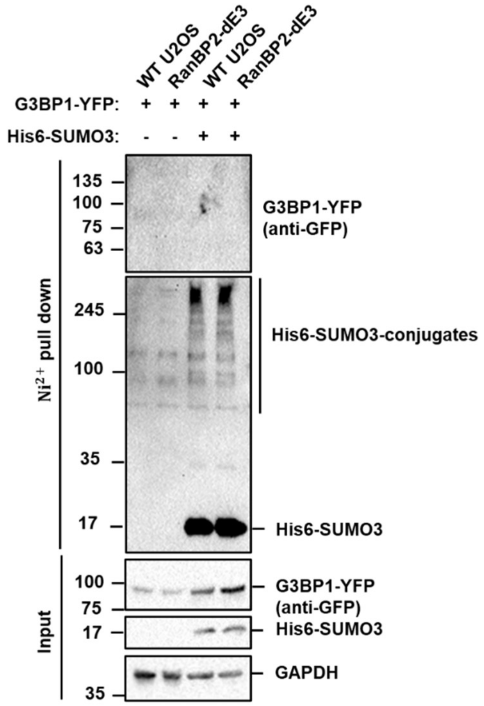

It should be noted that other groups have detected interactions between RanBP2 and G3BP2 (e.g., Ashikari et al., 2017), but there have been no similar reports between RanBP2 and G3BP1. So, while other groups have detected G3BP1 SUMOylation (for example, see Yuan et al., 2025), we decided to focus our efforts on G3BP2.

4. In Fig. 3D, G3BP2 shows up as a doublet. In the discussion, it is claimed that these are splicing products. However, His6-SUMO2-G3BP2 shows as a singlet. Are the authors claiming that only one isoform undergoes SUMOylation? Why?

We thank the reviewer for their astute observation. According to Ashikari et al, 2017, RanBP2-interaction requires a region that is not present in the short G3BP2 isoform. It is possible that the short isoform does not interact, or only interacts weakly, with RanBP2, thus explaining why only the larger isoform is robustly SUMOylated by RanBP2. We now discuss this in the text.

5. SUMOylation of a substrate should cause partial depletion of unconjugated substrate, whereas the opposite should also be true (e.g., SUMOylation of RanGAP, Fig. 1A). However, this does not appear to be clear from data presented in Figs. 3B and 3D. In fact, Fig. 3D appears to show depletions of both conjugated and unconjugated G3BP2 in the recovery phase.

We believe that the amount of SUMOylated G3BP2 is very low in the cells, since we do not detect the SUMOylated form when we blot for total G3BP2 in the cell lysate before nickel purification (input). Therefore, SUMOylation is not expected to cause a drastic change in the levels of unconjugated G3BP2. This is very different from RanGAP1, where a substantial proportion of RanGAP1 is SUMOylated and the SUMOylated form remains in a complex with RanBP2 which protects it from deSUMOylating enzymes. RanGAP seems to be the exception, for most proteins, only a very small fraction are SUMOylated at anyone given time. It is likely that SUMOylation of G3BP2 is a very transient event, which seems to be the case for many SUMOylated proteins.

6. What are the immunoreactivities of stress granules for SUMO1/2 in the cells used?

We thank the reviewers for prompting us to examine whether stress granules are immunoreactive for SUMO proteins. We now show that during oxidative stress, SUMO2/3, but not SUMO1, colocalizes with stress granules (Figure 3A-B) in U2OS cells, and this is partly dependent on RanBP2-mediated SUMOylation (Figure 3C-D).

Minor points: Abbreviations of G3BP1/2 need to be described in full.

We thank the reviewer for pointing this out. We now spell this out in the introduction.

Reviewer #2 (Remarks to the Author):

This report analyzes the relationship between RanBP2-dependent SUMOylation of G3BP2 and stress granule (SG) dynamics. The authors monitored SG formation and disassembly after sodium arsenite treatment and wash out in mutant U2OS cells that lack the SUMO ligase domain of RanBP2 (RanBP2-de3 cells). RanBP2-de3 cells show accelerated assembly of SGs upon sodium arsenite treatment and these SG persist longer than in control cells, although the peak number of SG was lower than controls (Figure 1). It has previously been demonstrated that RanBP2 depletion decreases p-body assembly; the authors show that RanBP2-de3 cells have fewer p-bodies, indicating that the steady-state level of p-bodies can be impacted by the mutation of RanBP2. Mass spec analysis of Ni2+ pull-down fractions from His6-SUMO2-expressing cells shows decreased levels of SG components G3BP1 and G3BP2 in the RanBP2-de3 cells compared to control U2OS cells, suggesting that these proteins may be SUMOylated in a RanBP2-dependent manner. The authors show that mutation of G3BP2 (K281R) decreases its SUMOylation levels, indicating this residue as a SUMO2 conjugation site. They further show that G3BP2 SUMOylation decreases with sodium arsenite treatment of control U2OS cells and then decreases further during the recovery period. Finally, the authors utilize U2OS cells lacking both G3BP1 and G3BP2 (ΔΔG3BP1/2) to express either G3BP2-HA or G3BP2-K281R-HA to measure their relative capacity for SG assembly. The authors observed that cells expressing G3BP2-K281R-HA had higher persistent SG levels in unstressed conditions, as well as modestly accelerated assembly of SGs after sodium arsenite treatment and SG that persisted longer than in G3BP2-HA-expressing cells during washout.

Taken together, these data suggest that while SUMOylation of K281 on G3BP2 by RanBP2 is not essential for SG formation or disassembly, it may contribute to the regulation of these

processes. As the authors note, it is counterintuitive that SUMOylation levels of G3BP2 are not stimulated by stress, but rather decrease upon stress and fall further during recovery (Figure 3E), so that there is not a simple correlation between G3BP2 SUMOylation and the level of SG assembly.

We have now repeated the SUMOylated kinetic experiments in U2OS cells not over-expressing Ubc9 and we observe different kinetics (Figure 3E). We now believe that the prior results may have been an artefact of Ubc9-overexpression. Our new data suggests that arsenite-treatment increases SUMOylation.

This manuscript would be far stronger if the authors could provide the logic for this observation and biochemical evidence of the regulatory mechanism underlying these phenomena.

We agree with the reviewer that a mechanistic understanding of how arsenite-treatment regulates SUMOylation would help to strengthen the paper. However, we believe that this is beyond the scope of the current study.

The manuscript could also be strengthened with a more substantial link between their observations on SG and p-bodies, which currently seems obscure.

We thank the reviewer for prompting us to examine the link between stress granules and P-bodies. We now provide data that RanBP2-mediated SUMOylation is required for the proper segregation of stress granules and P-bodies (Figure 5C-F). This raises the possibility that SUMOylation modulates the exchange of components between the two compartments.

Additional points to address:

• The conclusion that RanBP2 mediates SUMOylation of G3BP2 at K281R would be

substantially strenghtened by in vitro assays demonstrating SUMOylation with purified proteins to show the direct enzyme-substrate relationship. (Werner et al., 2009. PMID: 19107418)

We thank the reviewer for encouraging us to test whether RanBP2 directly SUMOylates G3BP2. We now show this using a recombinant SUMOylation system in *E. coli* (Figure 3E).

• In Figure 2, the author show that the number of p-bodies is lower in RanBP2-de3 cells, leading them to conclude that "the effects previously reported are likely due to changes in SUMOylation by RanBP2" (p.6). While this suggestion is consistent with the data, it is an over-interpretation. The RanBP2 mutations in RanBP2-de3 cells impact other aspects of RanBP2 function. For example, these mutations eliminate the RanGAP1 binding site at the nuclear pore, rendering it cytoplasmic and susceptible to de-SUMOylation (Zhu et al. 2009. PMID: 19285941). They also decrease the overall level of RanBP2 (Figure 1A,B). The authors should change the text to suitably acknowledge this ambiguity.

We thank the reviewer for bringing this up. We now address this important point at the start of the discussion where we state:

"Although it remains possible that the effects that we observe are due to changes in RanBP2 levels, or that the mutations in RanBP2 disrupt RanGAP1 localization to the nuclear pore, we favor the idea that the key change is SUMOylation per se, given that we show that RanBP2 directly SUMOylates G3BP2."

• Even if RanBP2 regulates p-body assembly primarily through SUMOylation, the findings in this manuscript seem preliminary because the targets may be unrelated to those involved in SG control. It is not clear how these findings coherently fit into the SG regulatory mechanism, but the manuscript would be strengthened if the logic of this link were clarified.

As described above, this point prompted us to more closely examine the relationship between stress granules and P-bodies (Figure 5C-F). Although we agree that this is unlikely due to G3BP2 SUMOylation, we believe that our findings are important enough to include in the current manuscript.

• The third column of the table in Figure 3A is not a ratio of the first two columns. It's not clear what it represents, so please clarify in the figure legend.

In this table (which is now Figure 2A), the number shown in the third column was the average ± SEM for the fold changes calculated for each individual experiment (the ratio of peptide counts in WT U2OS to RanBP2-dE3). We did not simply find the ratios of the averages since we believe it is important to demonstrate that the fold change itself is reproducible across experiments. We have now changed the figure legend to reflect this.

• Figure 3D,E shows that G3BP2 SUMOylation decreases with sodium arsenite treatment of control U2OS cells and then decreases further during the recovery period. Is this pattern altered in RanBP2-de3 cells, which still show G3BP2 SUMOylation at roughly half the level of untreated U2OS controls (Figure 3C)?

As described above, we now show that in RanBP2-dE3 cells, G3BP2 SUMOylation increases upon stress and remains high during recovery, though its levels were lower than in unmodified U2OS cells (Figure 3E-F).

RE: Manuscript #E25-03-0135R
TITLE: "RanBP2-dependent SUMOylation of G3BP2 inhibits formation, and promotes disassembly, of stress granules"

Dear Alex,

Thank you very much for the revised version of your manuscript and for addressing the points raised by the Reviewers. I sent the revised manuscript and your responses to their initial comments back to the Reviewers. They concur with my conclusion that the manuscript is now acceptable for publication. Congratulations to you and your colleagues on this excellent piece of work. Thank you for submitting your paper to MBoC. I hope we see more of your work in the future.

Best regards,

Rick

Richard Wozniak
Monitoring Editor
Molecular Biology of the Cell

Dear Dr. Palazzo:

Congratulations on the acceptance of your manuscript! Thank you for publishing your work in Molecular Biology of the Cell (MBoC).

Within 10 days, an unedited PDF of your manuscript will be published on MBoC in Press, an early-release journal version. The date your manuscript appears on this site is the official publication date.

Your copyedited and typeset manuscript will be scheduled for publication in the next available issue of MBoC. Once your paper is ready for review, our production team will notify you. In the summer of 2024, our production provider will introduce an online proofing solution, which will streamline the process of checking and validating author changes. This will result in improved typesetting quality, enhanced digital content, and a faster overall process.

We invite you to submit images related to your accepted manuscript to be considered for the journal cover.
Cover images must contain color, TIF Format
Size image at 7.4" wide x 5.85" deep
RGB and CMYK color (please submit two separate files, one in each format)
300 dpi resolution

Please also send a 1-paragraph description of the image that a general audience could understand. Include with the caption the name and institution of the individual(s) the image should be attributed to. Please provide this description in a Word file. You can send the file via email or by your favorite file transfer service to mboc@ascb.org.

Authors of Articles and Brief Communications are also encouraged to create a short video abstract to accompany their article when it is published. These video abstracts, known as Science Sketches, are up to 2 minutes long and will be published on YouTube and then embedded in the article abstract. Science Sketch Editors on the MBoC Editorial Board will guide you as you prepare your video. Information about how to prepare and submit a video abstract is available at www.molbiolcell.org/science-sketches. Please contact mboc@ascb.org if you are interested in creating a Science Sketch.

We look forward to publishing your paper in MBoC.
Sign up for MBoC's new content alerts here https://tinyurl.com/3bcsyk3e

Sincerely,

Eric Baker
Journal Production Manager
MBoC Editorial Office
mbc@ascb.org

Reviewer #1 (Remarks to the Author):

Although some concerns remain regarding the potential overreliance on ectopic overexpression of constructs that may introduce artefacts (as now acknowledged by the authors with the E2 ligase, Ubc9), the authors have generally and adequately addressed my comments. As it stands, this work provides a foundation for further research and for validating the interesting observations uncovered across biological and physiological systems and stress conditions.

Minor comments:

1. The authors do not appear to describe in the Methods (plasmid construct section) any plasmid related to RanBP2 dE3. The exact residue coordinates should also be specified.

2. The authors state that the use of a denaturing buffer (6 M guanidine-HCl) in pull down assays inactivates all deSUMOylating enzymes. While this may be true, the use of such a buffer-rather than non-denaturing buffers containing N ethylmaleimide (NEM) that more effectively preserve native protein conformations and interactions-raises potential concerns about whether guanidine-HCl could interfere with the interaction readouts described in this work. The authors should briefly comment in the manuscript on their choice of a denaturing buffer for pull down assays instead of non-denaturing buffers with NEM.

3. In Figure 2F, the RanBP2 band demonstrating its interaction with endogenous G3BP2 by co immunoprecipitation appears rather faint, with additional faint bands also visible in the control lanes.

Reviewer #2 (Remarks to the Author):

This is a significantly improved manuscript describing the relationship between RanBP2-dependent SUMOylation of G3BP2 and stress granule (SG) dynamics. Importantly, the authors have clarified the previously confusing relationship between G3BP2 SUMOylation and the kinetics of SG disassembly. Inclusion of a recombinant SUMOylation system in bacteria also provides better support for the conclusion that RanBP2 can act directly in G3BP2 SUMOylation. Additional changes in the text have clarified other concerns, and the manuscript should now be appropriate for publication.
